# *ent*-Clavilactone J and Its Quinone Derivative, Meroterpenoids from the Fungus *Resupinatus* sp.

**DOI:** 10.1021/acs.jnatprod.3c00174

**Published:** 2023-11-06

**Authors:** Karen Harms, Pathompong Paomephan, Thitiya Boonpratuang, Rattaket Choeyklin, Chuenchit Boonchird, Frank Surup

**Affiliations:** † Department Microbial Drugs, Helmholtz Centre for Infection Research, and German Centre for Infection Research (DZIF), Partner Site Hannover-Braunschweig, Inhoffenstrasse 7, 38124 Braunschweig, Germany; ‡ Department of Biotechnology, Faculty of Science, Mahidol University, 272 Thanon 4 Rama VI, Thung Phaya Thai, Ratchathewi, Bangkok 10400, Thailand; § National Biobank of Thailand (NBT), National Science and Technology Development Agency (NSTDA), 144 Thailand Science Park, Phahonyothin Road, Khlong Nueng, Khlong Luang, Pathum Thani 12120, Thailand; ∥ Biodiversity-Based Economy Development Office (Public Organization), The Government Complex Commemorating His Majesty the King’s 80th Birthday Anniversary 5 December 2007 Ratthaprasasanabhakdi Building, ninth Floor, Chaengwattana Road, Thung Song Hong, Lak Si, Bangkok 10210, Thailand; ⊥ Institute of Microbiology, Technische Universität Braunschweig, Spielmannstraße 7, 38106 Braunschweig, Germany

## Abstract

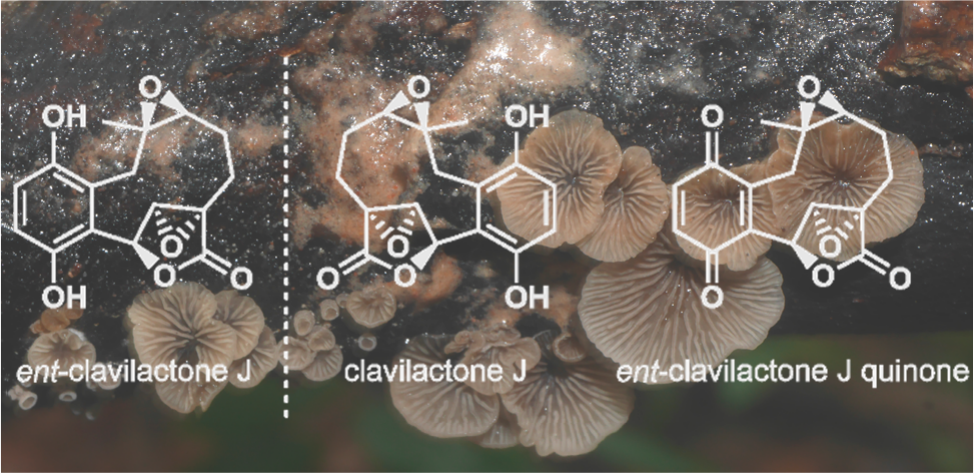

Metabolites **1** and **2**, isolated from cultures of the basidiomycete *Resupinatus* sp. BCC84615, collected in a tropical forest in northeastern Thailand, showed weak antibiotic activity against *Bacillus subtilis* and *Staphylococcus aureus* and cytotoxicity against cancer cell lines. Their planar structures were elucidated by high-resolution electrospray ionization mass spectrometry and NMR spectroscopy as clavilactone J, known from the basidiomycete *Ampulloclitocybe clavipes*, and its new 1,4-benzoquinone derivative. A detailed analysis of the ROESY correlations in **1** confirmed the recent revision of the relative configuration of clavilactone J. However, specific rotation and Cotton effects observed by electronic circular dichroism were contrary to those of the clavilactones; thus, we assigned a rare antipodal absolute configuration.

Fungal natural products continue to offer great potential for new innovative drugs.^1^ Novel species of Basidiomycota, the second largest group of fungi after the Ascomycota, collected from Thailand have proven to be a rich source of new metabolites in our screening program for new secondary metabolites.^2−6^ The present study deals with a Basidiomycota collected from northeastern Thailand and its secondary metabolites.

The strain *Resupinatus* sp. BCC84615 was identified by a comparison of morphological characteristics and sequencing of the 5.8S/ITS nrDNA region. A BLAST search in GenBank confirmed that the strain belongs to the genus *Resupinatus*. Based on its basidiocarps (Figure S1) and culture morphology, we classified this fungus as *Resupinatus* sp. The genus *Resupinatus* is distinguished by a spore-bearing surface folded into gills (lamellae) and small, virtually black fruiting bodies. *Resupinatus* and *Hohenbuehelia* have macromorphologies that are extremely similar. The unique characteristic that separates these two genera is the presence in *Hohenbuehelia* sp. of a gelatin layer in the cap tissue layer or pileus hymenium layer. This gelatin layer is absent in fungi belonging to *Resupinatus*. When examined under a microscope, the Thai specimens lacked a gelatin layer, which is consistent with the phylogenetic results indicating that they belong to the same clade as the genus *Resupinatus*.^7,8^ Members of *Resupinatus* have rarely been studied for secondary metabolites, and we know of only two studies describing secondary metabolites from the genus *Resupitatus*, including two 14-noreudesmane sesquiterpenoids described from *R. leightonhenii* and a bioactive germacrane sesquiterpene and a nerolidol derivative isolated from *R. leightonii*.^9,10^ Extracts of strain BCC84615 exhibited activity against *Bacillus subtilis*, and therefore, the strain was chosen for a detailed metabolite analysis.

The crude ethyl acetate extract of *Resupinatus* sp. BCC84615 contained only **1** and **2** as major metabolites (Figure S24), which were isolated subsequently by preparative HPLC. The molecular formula of **1** was deduced from the molecular ion cluster at *m*/*z* 305.1020 (M + H)^+^ in the high-resolution electrospray ionization mass spectrometry (HRESIMS) spectrum as C_16_H_16_O_6_, indicating 7 degrees of unsaturation. ^1^H and HSQC NMR data showed the presence of two aromatic methines, three oximethines, three methylenes, and one methyl. The ^13^C NMR spectrum furthermore indicated one carboxylic carbon (δ_C_ 171.9), four aromatic carbons (δ_C_ 149.1, 148.8, 125.9, and 120.3), and two oxygenated sp^3^-hybridized carbons (δ_C_ 61.3 and 60.6) devoid of bound protons. Based on the COSY correlations, the fragments H_2_-9/H_2_-10/H-11 and H-2/H-3 were assembled. The scaffold of **1** was elucidated using HMBC correlations (Tables S2, S3): correlations from methyl H_3_-15 to C-11/C-12/C-13; from H_2_-9 to C-7/C-8/C-10/C-11/C-16; from H-6 to C-4/C-5/C-7/C-8/C-14/C-16; and from H_2_-13 to C-1/C-5/C-11/C-12/C-14 permitted assembly of the γ-lactone connected to a 10-membered ring structure. Correlations from H-2 to C-1/C-14, H-3 to C-4/C-5, H-6 to C-4/C-5, and H_2_-13 to C-1/C-14 suggested attachment of the tetrasubstituted aromatic ring to the main ring. The C-7/C-8 and C-11/C-12 epoxide moieties were indicated by the deshielding of H-7 (δ_H_ 3.98) and H-11 (δ_H_ 2.77) and explained the remaining two degrees of unsaturation. Although **1** was not included in the database Dictionary of Natural Products^11^ or SciFinder^N^,^12^ the structure of **1** was described by Hou et al. in 2022 as clavilactone J.^13^ Very recently, Novitskiy and Kutateladze revised the configuration of clavilactone J using a DFT computational NMR method.^14^ In our study, we back up this structural revision based on ROESY data. In the ROESY NMR spectrum, key correlations were observed between H-7/H_3_-16 and H-7/H-11 (Figure 1), confirming a 6R*,7R*,8R*,11R*,12S* relative configuration, since both H-11 and H_3_-16 are oriented on the same face. However, our measured specific rotation, [α]^20^_D_ = −22 (*c* 0.1, MeOH), was identical in magnitude but opposite in sign to that previously reported ([α]^25^_D_ = +22 (*c* 0.05, MeOH). Additionally, the electronic circular dichroism (ECD) spectrum of **1** (Figure 2) is a mirror image of that of clavilactone J described by Hou and colleagues.^13^ Consequently, compound **1** was unambiguously assigned as *ent*-clavilactone J.

**Figure 1:**
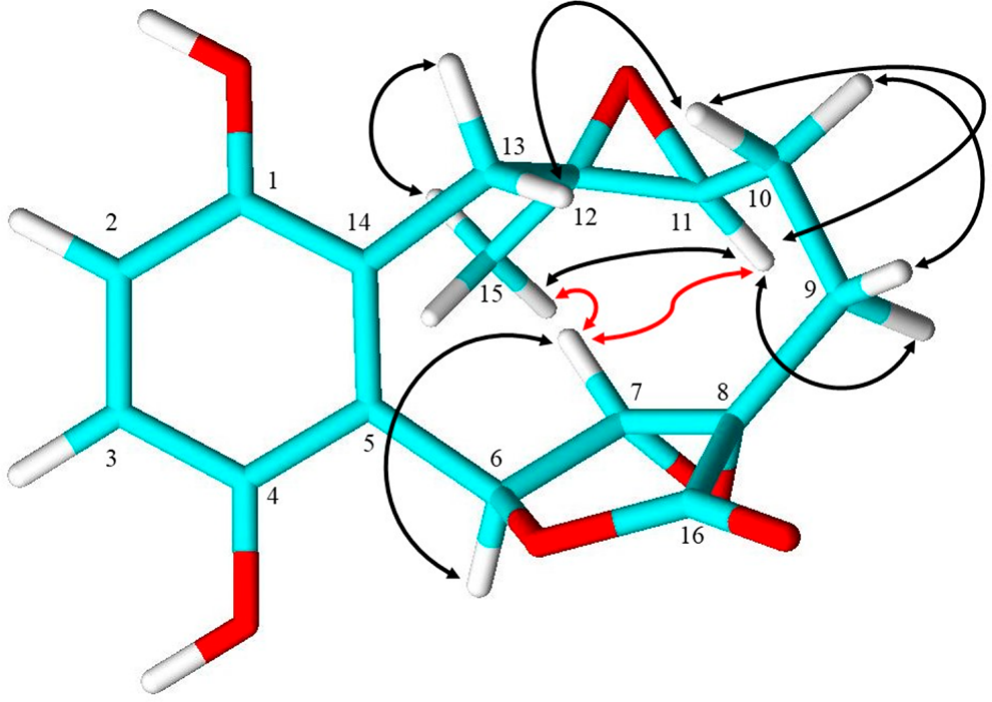
ROESY correlations in *ent*-clavilactone J (**1**). Key ROESY correlations between H-7/H_3_-16 and H-7/H-11 in red confirm a 6R*,7R*,8R*,11R*,12S* relative configuration.

**Figure 2:**
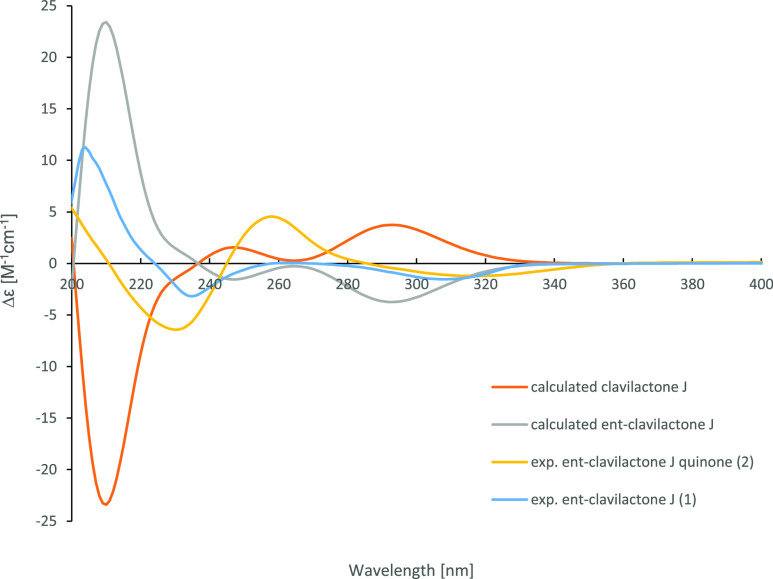
Experimental ECD spectra of **1** and **2**; calculated ECD spectra of clavilactone J and *ent-*clavilactone J.^14^

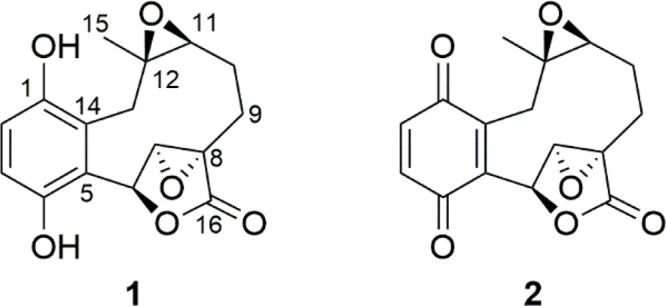



Metabolite **2** was isolated as a solid with the molecular formula C_16_H_14_O_6_, corresponding to an additional degree of unsaturation. The ^1^H and HSQC NMR spectra of **2** were highly similar to those of **1**. However, key differences were observed for aromatic ring carbons C-1, C-2, C-3, C-4, C-5, and C-14 in the ^13^C NMR spectrum. In particular, the chemical shifts of C-1 and C-4 indicate a *para*-quinone system. Therefore, **2** was identified as the 1,4-benzoquinone derivative of **1** and named *ent*-clavilactone J quinone.

The clavilactones are a family of meroterpenoids containing a constrained 10-membered ring fused to a 2,3-epoxy-γ-lactone and a benzoquinone or hydroquinone from cultures of the edible mushroom *Ampulloclitocybe clavipes* (synonym *Clitocybe clavipes*).^15,16^ Since the initial isolation of clavilactones A–C,^17^ derivatives clavilactone D,^18,19^ E,^20^ F,^21^ G–I,^22^ and J and K^13^ have been isolated from the same producing species. The derivatives differ from parental clavilactones A and B by hydroxylation or amination at different locations (Figure S23). To evaluate the taxonomic relationship of strain BCC84615 to *Resupinatus* and *Ampulloclitocybe*, a maximum-likelihood phylogenetic tree was generated showing the relationship between *Resupinatus* and closely related genera like *Hohenbuehelia*. The *Ampulloclitocybe* sequence was included to visualize the distance from the genus *Resupinatus*. In the resulting tree, *Resupinatus* and *Ampullocitocybe* are substantially separated, allowing us to use *Ampullocitocybe*, which is from a different family, as the outgroup for this population’s analysis. In other words, **1** and its previously reported enantiomer clavilactone J originate from distinct organism families, a highly surprising result (Figure S2).

In addition to antibacterial and antifungal activities, clavilactones A, B, and D are epidermal growth factor receptor tyrosine kinase inhibitors.^21^ Furthermore, *seco*-clavilactone B is an actin polymerization inhibitor.^22^

For **1**, we observed weak antibiotic activity against Gram-positive bacteria (*S. aureus*, *B. subtilis*) in our panel of test organisms,^23^ whereas Gram-negative bacteria and fungi were not affected (Table 1). *ent*-Clavilactone J, and to a lesser extent, the quinone form, exhibited antiproliferative effects against various cell lines (Table 2). This pattern of activity contrasts with that of Wang et al., who observed stronger anti-hepatoma activities of related 1,4-benzoquinone compounds compared to 1,4-dihydroxybenzene derivatives.^24^

**Table 1:** Minimum Inhibitory Concentration (MIC, μg/mL) of **1** and **2** against Several Bacterial and Fungal Strains^a^

test organism	strain number	**1**	**2**	positive control
*Bacillus subtilis*	DSM 10	17	67	8.3
*Mycolicibacterium smegmatis*	ATCC 700084	–	–	1.7
*Staphylococcus aureus*	DSM 346	–	67	1.7; 0.21
*Acinetobacter baumanii*	DSM 30008	–	–	0.26; 0.53
*Chromobacterium violaceum*	DSM 30191	–	–	0.42; 1.7
*Escherichia coli*	DSM 1116	–	–	1.7
*Pseudomonas aeruginosa*	DSM 19882	–	–	0.42; 0.21
*Mucor hiemalis*	DSM 2656	–	–	4.2; 8.3
*Candida albicans*	DSM 1665	–	–	8.3
*Rhodotorula glutinis*	DSM 10134	–	–	2.1; 4.2
*Schizosaccharomyces pombe*	DSM 70572	–	–	4.2; 8.3
*Wickerhamomyces anomala*	DSM 6766	–	–	8.3

a ATCC: American Type Culture Collection; DSM: Leibniz-Institut DSMZ—German Collection of Microorganisms and Cell Cultures GmbH. – means no inhibition observed under test conditions.

**Table 2:** Cytotoxicity of **1** and **2** against Mammalian Cell Lines (IC_50_ in μM)

			IC_50_ [μM]			
cell line	type	number^a^	**1**	epothilone B^b^	**2**	epothilone B^b^
L929	mouse fibroblasts	ACC 2	2.1	4.7 × 10^–4^	6.0	4.3 × 10^–4^
KB 3.1	human endocervical adenocacinoma (AC)	ACC 158	6.7	3.4 × 10^–5^	22	7.3 × 10^–5^
A431	human squamous AC	ACC 91	3.5	5.1 × 10^–5^	6.5	6.5 × 10^–5^
A549	human lung carcinoma	ACC 107	19	6.7 × 10^–5^	57	5.3 × 10^–5^
MCF-7	human breast AC	ACC 115	1.9	3.0 × 10^–5^	4.2	8.3 × 10^–5^
PC-3	human prostate AC	ACC 465	18	9.5 × 10^–5^	19	2.8 × 10^–4^
SK-OV-3	human ovary AC	n/a	47	2.6 × 10^–4^	30	1.8 × 10^–4^

a ACC: Leibniz-Institut DSMZ—German Collection of Microorganisms and Cell Cultures GmbH.

b Positive control (1 mg/mL).

Chiral natural products are usually produced in nature in an optically pure form. Only occasionally are both enantiomers formed.^25^ As an exception, many chiral monoterpenes in plants are produced in both enantiomeric forms, often by the same species. These enantiomeric monoterpenes display unique biological activities, oftentimes with each enantiomer exhibiting distinct biological properties. However, this phenomenon is rare among fungal terpenoids. In the case of the enantiomeric clavilactones, the opposite configuration is not generated by the terpene cyclase itself but in the course of subsequent oxidation reactions. In the proposed biosynthesis (Scheme 1), the achiral intermediate wigandolis is formed via the cyclization of geranylhydroquinone. Further oxidations, presumably catalyzed by P450 oxidases, lead to arnebinol A (which is known to coexist with clavilactone A in the plant *Arnebia euchroma*(25)) and to other members of the clavilactone family.

**Scheme 1:**
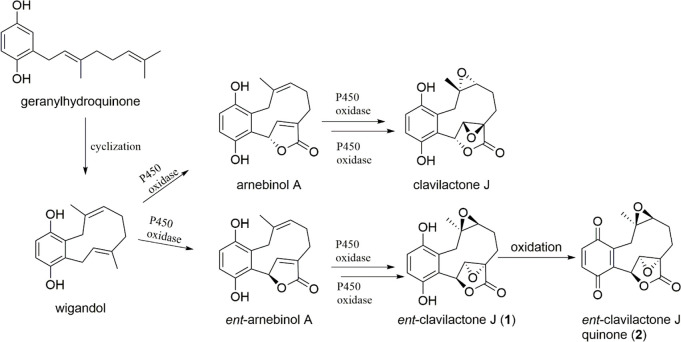
Proposed Biosynthesis of Clavilactone J, *ent*-Clavilactone J (**1**), and *ent*-Clavilactone J Quinone (**2**)

In the case of *ent*-clavilactones **1** and **2**, these oxidation reactions take place from the opposite direction and require three individual, antipodal steps. It will be interesting to discover how the different action of P450 enzymes is generating the different enantiomers of the clavilactones.

## Experimental Section

### General Experimental Procedures

Optical rotations were measured using an Anton Paar MCP-150 polarimeter with a 100 mm path length and sodium D line at 589 nm. UV spectra were measured on a Shimadzu UV/vis 2450 spectrophotometer using methanol (Uvasol, Merck) as a solvent. ECD spectra were measured with a J-815 spectropolarimeter (Jasco) by using methanol as a solvent. The spectral data are combined in Figure 2). 1D and 2D NMR spectra were measured on a Bruker 700 MHz Avance III spectrometer equipped with a 5 mm TCI cryoprobe and a Bruker Avance III 500 spectrometer. NMR data were referenced to residual solvent peaks (δ_H_ 7.27 ppm, δ_C_ 77.0 ppm for CDCl_3_; δ_H_ 2.50 ppm, δ_C_ 39.51 ppm for DMSO-*d*_6_).

HPLC-DAD/MS data were measured using an amaZon speed ETD (electron transfer dissociation) ion trap mass spectrometer (Bruker Daltonics) and were measured in positive and negative ion modes simultaneously. The HPLC system was run with a C18 Acquity UPLC BEH (Waters) column with mobile phases of water (H_2_O, solvent A) and acetonitrile (MeCN, solvent B) each supplemented with 0.1% formic acid (FA). The following gradient conditions were used: 5% B for 0.5 min, increasing to 100% B in 20 min, hold at 100% B for 10 min, with a flow rate of 0.6 mL/min, and UV/vis detection at 200–600 nm.

HRESIMS data were recorded on a MaXis ESI-TOF (electrospray ionization-time-of-flight) mass spectrometer (Bruker GmbH) coupled to an Agilent 1260 series HPLC-UV system and equipped with a C18 X-Bridge, 100 × 2.1 mm, 3.5 μm d_p_ column (Waters); DAD-UV detection at 200–600 nm; solvent A = 5% MeCN, 95% water + 5 mM NH_4_CH_3_COO, pH 5.5 (40 μL CH_3_COOH/L) and solvent B = 95% MeCN, 5% water + 5 mM NH_4_CH_3_COO, pH 5.5 (40 μL CH_3_COOH/L) as a modifier; flow rate 0.3 mL/min, 40 °C, gradient elution from 10% to 100% B over 30 min and holding at 100% B for 10 min. A Bruker Compass DataAnalysis ver. 4.4 was used to analyze the data, including determining the molecular formula using the Smart Formula algorithm (Bruker Daltonics).

### Fungal Material

In May 2017, *Resupinatus* sp. was collected from an unnamed decaying dicotyledon twig in Dong Yai Community Forest, Amnat Charoen Province, located in the northeastern part of Thailand. Collections were made on behalf of the Plant Genetics Conservation Project under the Royal Initiative of Her Royal Highness Maha Chakri Sirindhorn (RSPG). The voucher specimen was deposited in the BIOTEC Bangkok Herbarium & Fungarium with the designation BBH42540. The fungal strain has been officially registered for preservation at both the National Biobank of Thailand (NBT) under the designation NBTM1549 and the BIOTEC Culture Collection under the accession number BCC84615. The genetic sequences have been submitted to the accessible GenBank under accession number OL672739.

### Fermentation and Extraction

The fungus was grown at 25 °C on yeast malt (YM) agar (10 g/L malt extract, 4 g/L yeast extract, 4 g/L d-glucose, and 20 g/L agar, adjusted to pH 6.3). Once established, the mycelia were cut into discs with a cork borer. Five discs were placed into each of seven 500 mL Erlenmeyer flasks containing 250 mL of MGP-medium (10 g/L d-glucose, 20 g/L maltose, 2 g/L soy peptone, 1 g/L yeast extract, 1 g/L KH_2_PO_4_, 0.5 g/L MgSO_4_ × 7 H_2_O, and 1 mL/L each of stock solution of 10 mM FeCl_3_, 1,78 g/L ZnSO_4_, 0.1 M CaCl_2_). Cultures were agitated at 140 rpm at 23 °C for 3 weeks.

The mycelium was separated from the supernatant with a paper filter, and both the mycelium and supernatant were extracted separately. The mycelium was covered with acetone and sonicated in an ultrasonic bath at 40 °C for 30 min, then filtered and sonicated again. The two acetone extracts were combined and evaporated in vacuo at 40 °C. The remaining aqueous residue was extracted twice with one volume of ethyl acetate each time. The supernatant was also extracted twice with one volume of ethyl acetate each time. Both ethyl acetate extracts were dried over sodium sulfate and evaporated in vacuo at 40 °C to dryness. The yield of the mycelium extract was 30 mg, and that of the supernatant extract was 124 mg.

### Isolation of Compounds **1** and **2**

A 25 mg portion of supernatant extract was separated on a PLC 2250 preparative HPLC system (Gilson) equipped with a Gemini 10u C18 110 Å column (250 × 21.20 mm, 10 μm; Phenomenex) as the stationary phase and in the following conditions: solvent A = H_2_O, solvent B = MeCN; flow rate: 20 mL/min, fractionation: 10 mL, gradient: 5% to 24% B over 12 min, 24% to 30% B over 25 min, 30% to 37% B over 7 min, 37% to 100% in 20 min, and a hold at 100% B for 15 min. This yielded the pure fractions of **1** (2 mg, *t*_R_ = 24–26 min) and **2** (5 mg, *t*_R_ = 37–39 min).

#### *ent*-Clavilactone J (**1**):

amorphous solid; [α]^20^_D_ = −22 (*c* 0.1, MeOH); UV (MeOH) λ_max_ (log ε) 310 (3.6), 202 (4.5); ECD (16 μM, MeOH) λ_max_ (Δε) 204 (+11.3), 235 (−3.2), 263 (+0.1), 308 (−1.5), 336 (−0.1) nm; ^1^H NMR (CDCl_3_, 700 MHz) δ 6.79 (d, *J* = 8.6 Hz, H-3), 6.69 (d, *J* = 8.6 Hz, H-2), 6.35 (d, *J* = 0.7 Hz, H-6), 3.98 (br s, H-7), 3.41 (d, *J* = 15.7 Hz, H_a_-13), 2.86 (m, H_a_-9), 2.77 (dd, *J* = 9.5, 4.2 Hz, H-11), 2.45 (m, H_a_-10), 2.25 (d, *J* = 15.7 Hz, H_b_-13), 1.57 (m, H_b_-9), 1.56 (m, H_b_-10), 1.13 (s, H_3_-15); ^13^C NMR (CDCl_3_, 175 MHz) δ 171.5 (C, C-16), 149.2 (C, C-1), 148.8 (C, C-4), 125.9 (C, C-5), 120.3 (C, C-14), 118.0 (CH, C-3), 115.5 (CH, C-2), 74.1 (CH, C-6), 64.9 (CH, C-11), 63.1 (CH, C-7), 61.3 (C, C-8), 60.6 (C, C-12), 26.6 (CH_2_, C-13), 24.5 (CH_2_, C-10), 22.3 (CH_2_, C-9), 21.4 (CH_3_, C-15); ESI-MS *m*/*z* 305.01 (M + H)^+^ and 327.03 (M + Na)^+^; HRESIMS *m*/*z* 327.0854 (M + Na)^+^ (calcd for C_16_H_16_O_6_Na 327.0839) and *m*/*z* 305.1033 (M + H)^+^ (calcd for C_16_H_17_O_6_, 305.1020).

#### *ent*-Clavilactone J quinone (**2**):

amorphous solid; [α]^20^_D_ = +69 (*c* 0.1, MeOH); UV (MeOH) λ_max_ (log ε) 250 (4.0); ECD (17 μM, MeOH) λ_max_ (Δε) 195 (+9.7), 230 (−6.4), 258 (+4.6), 317 (−1.2), 376 (+0.1) nm; ^1^H NMR (CDCl_3_, 500 MHz) δ 6.99 (d, *J* = 10.0 Hz, H-2), 6.96 (d, *J* = 10.0 Hz, H-3), 6.10 (d, *J* = 0.8 Hz, H-6), 3.94 (d, *J* = 0.8 Hz, H-7), 3.48 (d, *J* = 14.7 Hz, H_a_-13), 2.84 (br dd, *J* = 13.8, 6.0 Hz, H_a_-9), 2.75 (dd, *J* = 10.1, 3.8 Hz, H-11), 2.50 (m, H_a_-10), 2.11 (d, *J* = 14.7 Hz, H_b_-13), 1.58 (ddd, *J* = 14.2, 13.8, 1.8 Hz, H_b_-9), 1.50 (m, H_b_-10), 1.13 (s, H_3_-15); ^13^C NMR (CDCl_3_, 125 MHz) δ 186.5 (C, C-1), 184.5 (C, C-4), 170.4 (C, C-16), 146.0 (C, C-14), 137.0 (C, C-5), 136.8 (CH, C-2), 136.4 (CH, C-3), 70.7 (CH, C-6), 64.8 (CH, C-11), 61.9 (CH, C-7), 60.4 (C, C-8), 60.2 (C, C-12), 25.5 (CH_2_, C-13), 24.5 (CH_2_, C-10), 21.9 (CH_2_, C-9), 21.7 (CH_3_, C-15); ESI-MS: *m*/*z* 301.98 (M – H)^−^ and 325.01 (M + Na)^+^; HRESIMS *m*/*z* 325.0683 (M + Na)^+^ (calcd for C_16_H_14_O_6_Na 325.0683) and *m*/*z* 303.0862 (M + H)^+^ (calcd for C_16_H_15_O_6_, 303.0863).

## Supporting Information Available

Additional figures and tables (PDF)
Raw NMR data(ZIP)
