# Supplementary material for: ent-Clavilactone J and Its Quinone Derivative, Meroterpenoids from the Fungus Resupinatus sp
Source: J Nat Prod. 2023 Nov 6;86(11):2580–4. doi: 10.1021/acs.jnatprod.3c00174 (PMC10683060; doi:10.1021/acs.jnatprod.3c00174)
Supplement: Supplementary file 1 — np3c00174_si_001.pdf [file np3c00174_si_001.pdf]

Supplementary material

ent-clavilactone J and its quinone derivative, meroterpenoids from the fungus *Resupinatus* sp.

Karen Harms[1†], Pathompong Paomephan[1,2†], Thitiya Boonpratang[3], Rattaket Choeyklin[3,4], Chuenchit Boonchird[2] and Frank Surup[1,5,]*

1 Department Microbial Drugs, Helmholtz Centre for Infection Research, Inhoffenstrasse 7, 38124 Braunschweig, Germany and German Centre for Infection Research (DZIF), Partner Site Hannover-Braunschweig, Inhoffenstraße 7, 38124 Braunschweig, Germany

2 Department of Biotechnology, Faculty of Science, Mahidol University, 272 Rama VI Road, Thung Phaya Thai, Ratchathewi, Bangkok 10400, Thailand

3 National Biobank of Thailand (NBT), The National Science and Technology for Development Agency (NSTDA), Khlong Luang, Pathum Thani 12120 Thailand

4 Biodiversity-Based Economy Development Office (Public Organization) (BEDO), Lak Si, Bangkok 10210 Thailand

5 Institute of Microbiology, Technische Universität Braunschweig, Spielmannstraße 7, 38106 Braunschweig, Germany

† These authors contributed equally to this work

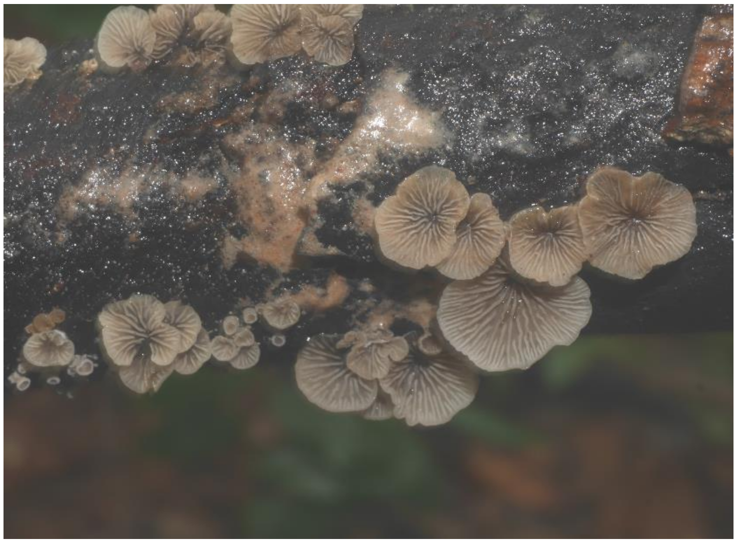

Figure S1. The basidiocarps of *Resupinatus* sp. on an unidentified rotting tree trunk.

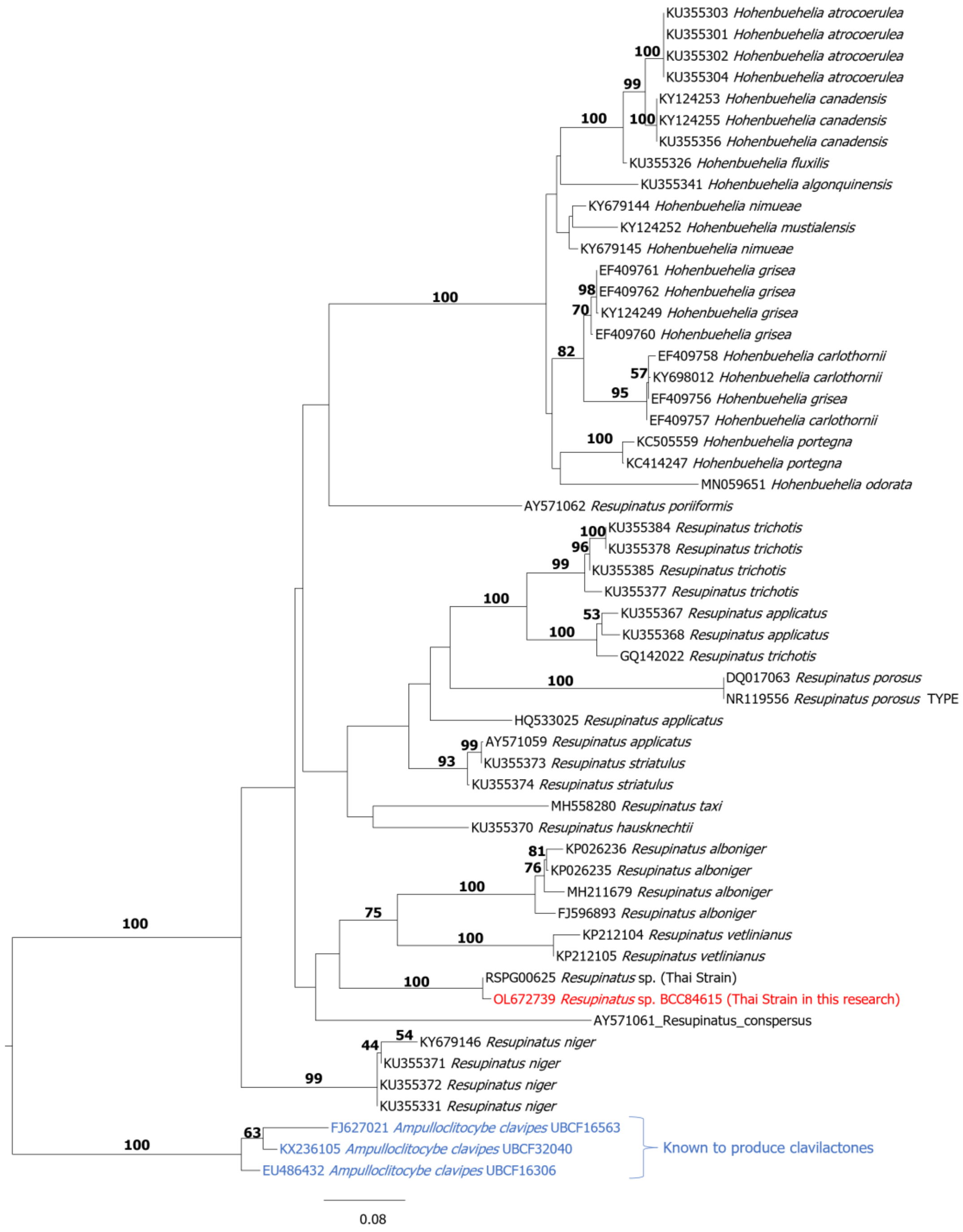

Figure S2. Maximum-likelihood *Resupinatus* tree. Red indicates sequences retrieved from mushroom culture strain in this study (OL672739 is a GenBank code which designated as BCC84615 where deposited in BIOTEC Culture Collection). Bootstraps less than 50 were omitted. Taxa equals 55 and length equals 1018. The species *Ampulloclitocybe clavipes* is utilized for our group.

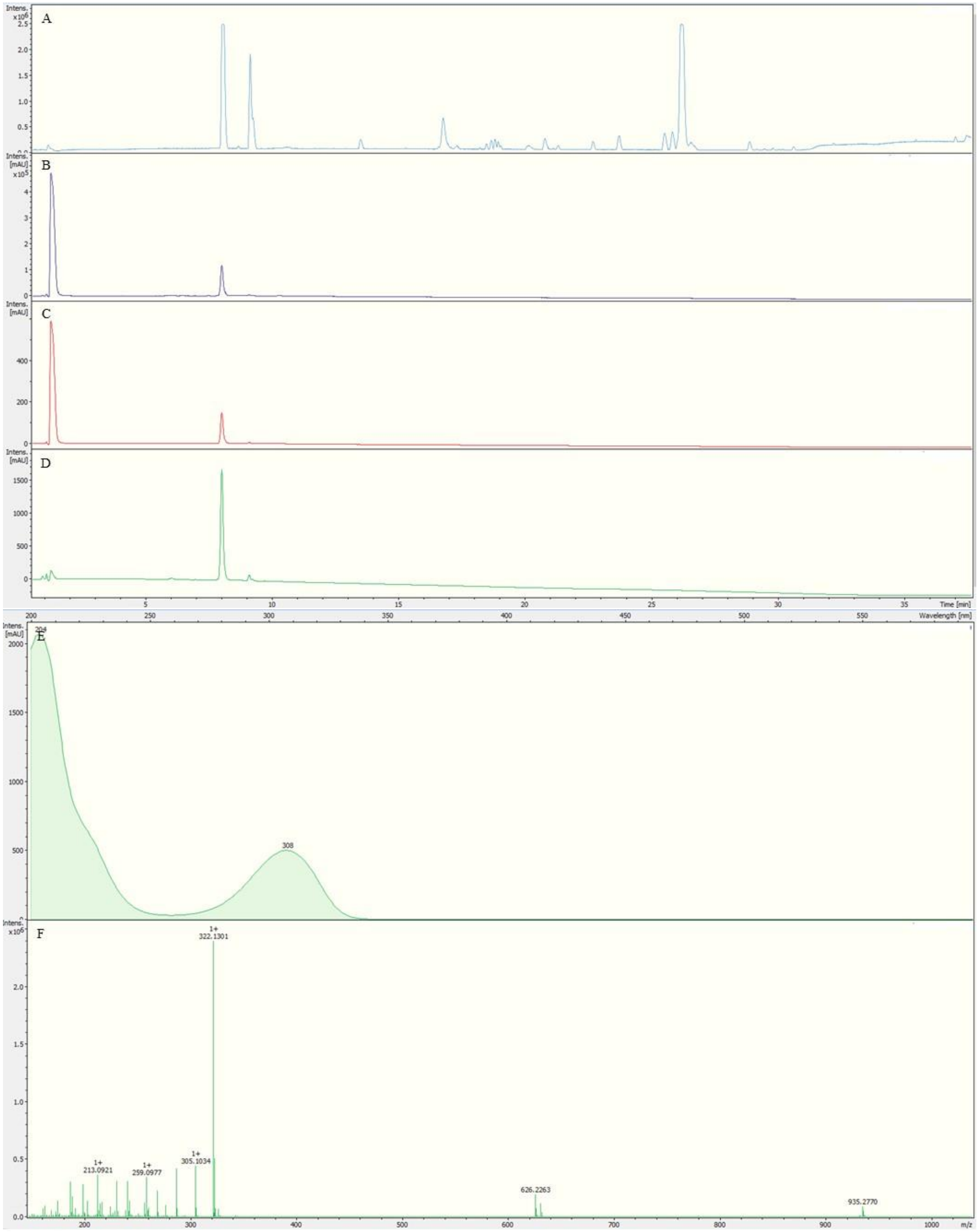

Figure S3. HRESIMS of *ent*-clavilactone J (**1**) isolated from MGP medium. A is the positive mass spectra. B, C and D are the chromatograms at 200-600 nm, 200 nm and at 210nm. E and F are the extracted UV spectrum and the mass spectrum of **1**.

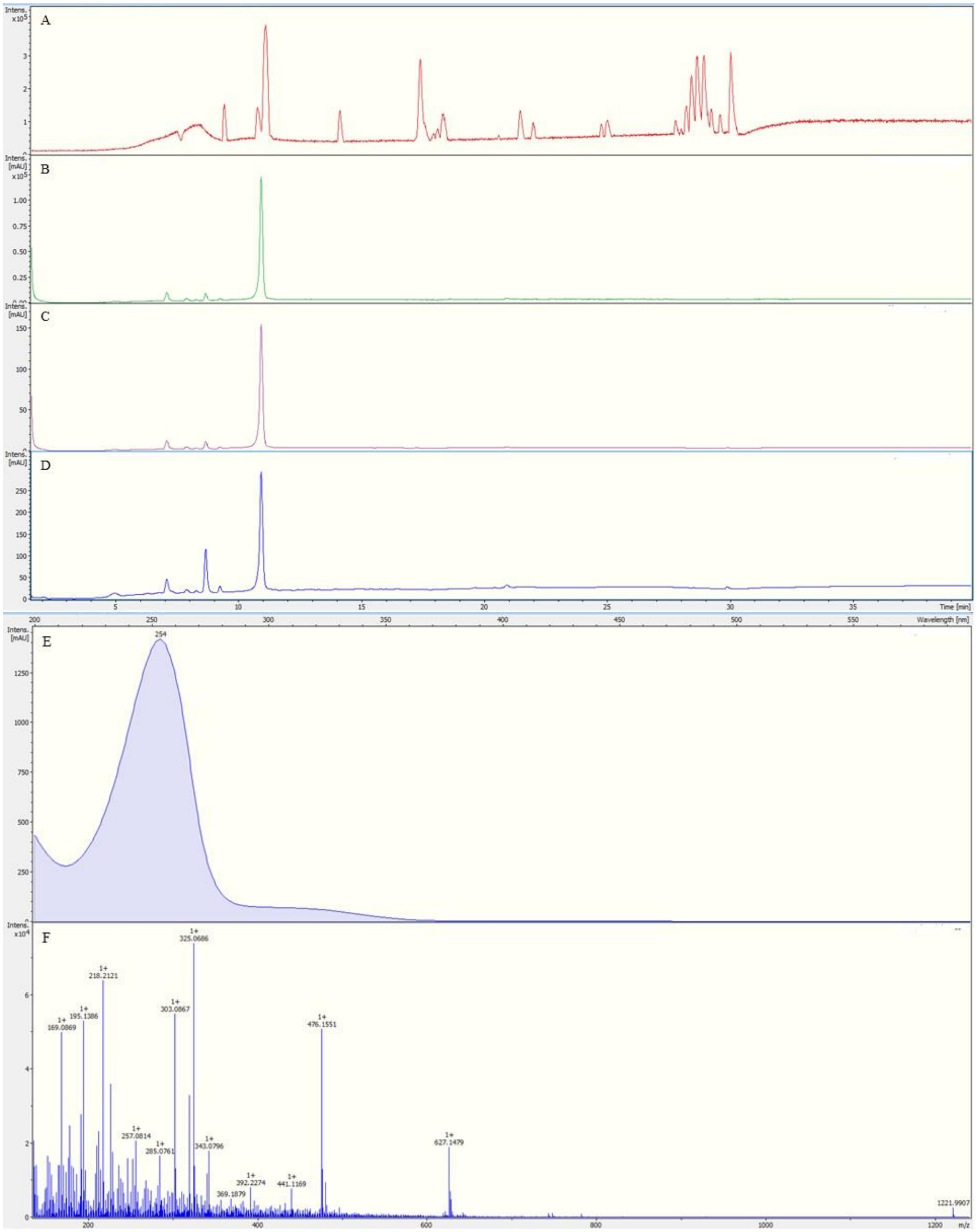

Figure S4. HRESIMS of *ent*-clavilactone J quinone (**2**) isolated from MGP medium. A is the positive mass spectra. B, C and D are the chromatograms at 200-600 nm, 200 nm and at 210nm. E and F are the extracted UV spectrum and the mass spectrum of **2**.

Table S1. NMR data (^{1}H, 700 MHz, ^{13}C 175 MHz) of *ent*-clavilactone J (**1**) in $CHCl_3$-*d*.

position	δ_C type	δ_H (mult., J in Hz)	COSY	ROESY	C to H HMBC
1	149.2, C	-	-	-	-
2	115.5, CH	6.69, d (8.6)	-	15, 13''	1, 3, 4, 5, 14
3	118.0, CH	6.79, d (8.6)	-	15	1, 2, 4, 5
4	148.8, C	-	-	-	-
5	125.9, C	-	-	-	-
6	74.1, CH	6.35, d (0.7)	9', 7	7	4, 5, 7, 8, 14, 16
7	63.1, CH	3.98, br s	6, 9'	6, 9'', 11, 13'', 15	6, 8, 16
8	61.3, C	-	-	-	-
9	22.3, CH_2	2.86, m	6, 7, 10', 10''	9'', 10', 10'', 13''	7, 8, 10, 16
		1.57, m	10'	7, 9',10', 11, 13''	8, 10, 11, 16
10	24.5, CH_2	2.45, m	9',9'', 11	9', 9'', 11, 15	9, 11, 12, 16
		1.56, m	9', 11	9', 11, 13''	9
11	64.9, CH	2.77, dd (9.5, 4.2)	10', 10''	7, 9'', 10', 10'', 13'', 15	10, 15
12	60.6, C	-	-	-	-
13	26.6, CH_2	3.41, d (15.7)	13'', 15	13'', 15	1, 5, 11, 12, 14
		2.25, br d (15.7)	13'	2, 7, 9', 9'', 10'', 11, 13', 15	1, 5, 11, 12, 14, 15
14	120.3, C	-		-	-
15	21.4, CH_3	1.13, s	13'	2, 3, 7, 10', 11, 13', 13''	11, 12, 13
16	171.5, C	-		-	-

Table S2. NMR data (^{1}H, 500 MHz, ^{13}C 125 MHz) of *ent*-clavilactone J (**1**) in DMSO-d_6.

position	δ_C type	δ_H (mult., J in Hz)	COSY	ROESY	C to H HMBC
1	149.2, C	-	-	-	-
2	117.8, CH	6.84, d (8.6)	3	1OH	1, 5, 13, 14
3	115.0, CH	6.73, d (8.7)	2	4OH	4, 5, 6, 14
4	149.0, C	-	-	-	-
5	118.9, C	-	-	-	-
6	74.6, CH	6.16, s	-	7	4, 5, 7, 8, 14, 16
7	62.7, CH	4.47, s	-	6, 9", 11, 15	6, 8, 16
8	61.0, C	-	-	-	-
9	21.5, CH_2	2.57, m	9", 10"	9", 10"	7, 8, 10, 16
		1.60, m	9", 10', 10"	9', 9", 10', 10"	8, 10, 11, 16
10	24.1, CH2	2.35, m	9', 9", 10", 11	9", 10", 11	11
		1.22, m	9", 10", 11	9', 10', 13"	8, 9, 11
11	63.5, CH	2.78, dd (10.3, 3.7)	10', 10"	7, 9", 10', 15	10, 12, 15
12	60.2, C	-	-	-	-
13	26.0, CH_2	324, m	13"	13"	1, 5, 11, 12, 14
		1.94, m	13'	9", 10", 13'	1, 5, 11, 12, 14, 15
14	124.4, C	-	-	-	-
15	21.2, CH_3	1.00, s	-	7, 11	11, 12, 13
16	171.9, C	-	-	-	-
1OH	OH	9.23, s	-	2	1, 2, 14
4OH	OH	9.49, s	-	3	4, 5, 14

Table S3. . NMR data (^{1}H, 500 MHz, ^{13}C 125 MHz) of *ent*-clavilactone J quinone (**2**) in $CDCl_3$.

**position**	**δ_C type**	**δ_H (mult., J in Hz)**	**COSY**	**ROESY**	**C to H HMBC**
1	186.5, C				
2	136.8, CH	6.99, d (10.0)	-	-	4, 14
3	136.4, CH	6.96, d (10.0)	-	-	1, 5, 6
4	185.5, C				
5	137.0, C				
6	70.7, CH	6.10, d (0.8)	9'	7', 13"	4, 5, 7, 8, 14, 16
7	61.9, CH	3.94, d (0.8)	9'	6, 9", 11, 15	5, 6, 8, 9, 16
8	60.4, C				
9	21.9, CH_2	2.84, br dd (13.8, 6.0)	10', 9"	9", 10', 10"	7, 8. 10, 11, 16
		1.58, ddd(14.2, 13.8, 1.8)	9'	7, 9',10'	8, 10, 11, 16
10	24.5, CH_2	2.50, m	9', 10", 11	9', 10", 11	8, 9, 11
		1.50, m	9',10', 13"	9',10'	8, 9, 11
11	64.8, CH	2.75, dd (10.1, 3.8)	10', 15	7, 10', 15	10, 15
12	60.2, C				
13	25.5, CH_2	3.48, br d (14.7)	13"	13", 15	1, 5, 11, 12, 14
		2.11, br d (14.7)	13', 15	6, 10", 13'	1, 5, 11, 12, 14, 15
14	146.0, C				
15	21.7, CH_3	1.13, s	13"	7, 10', 11, 13', 13"	11, 12, 13, 14
16	170.4, C				

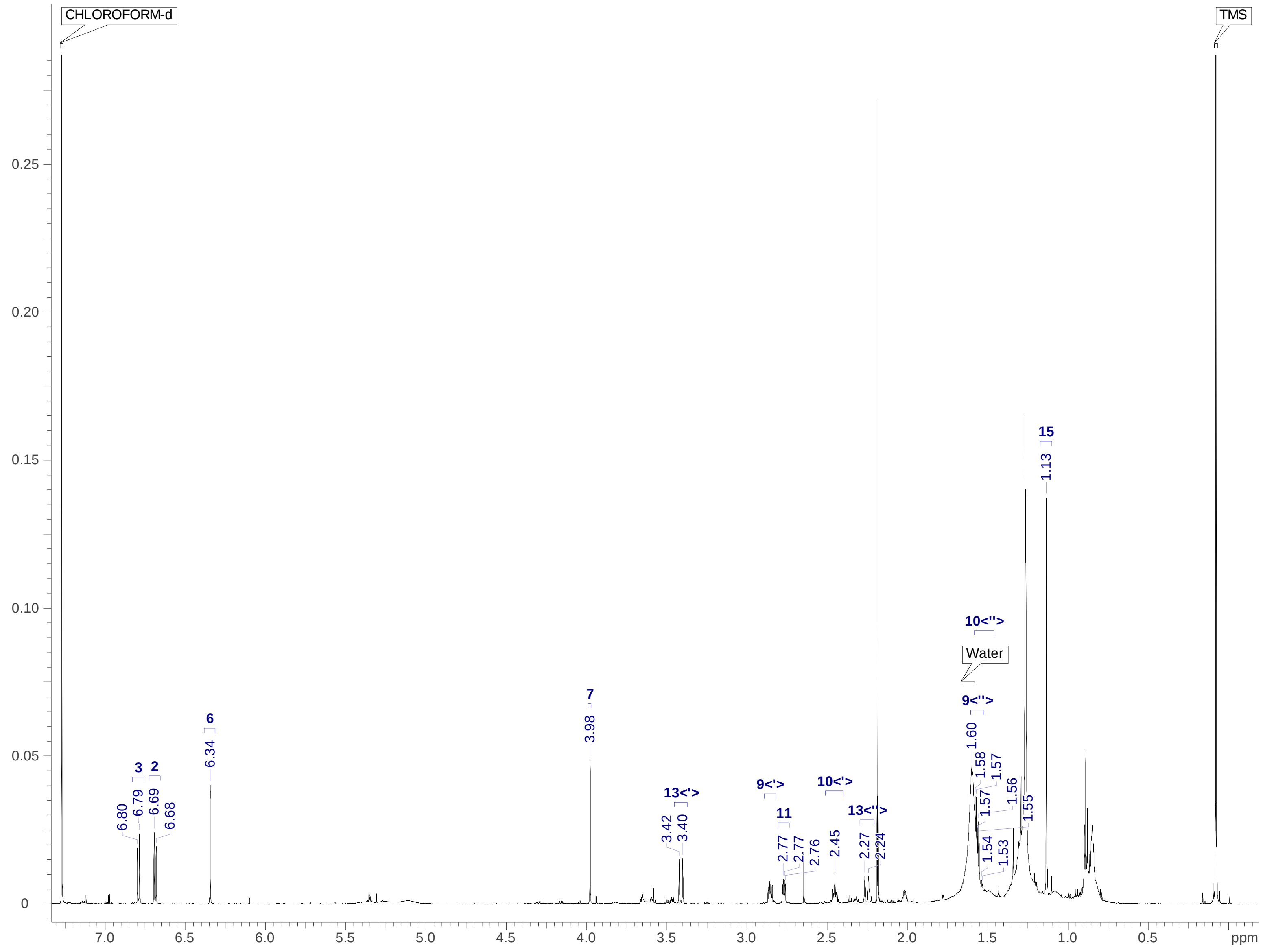

Figure S5. ^{1}H NMR spectrum (700 MHz) *ent*-clavilactone J in $CDCl_3$

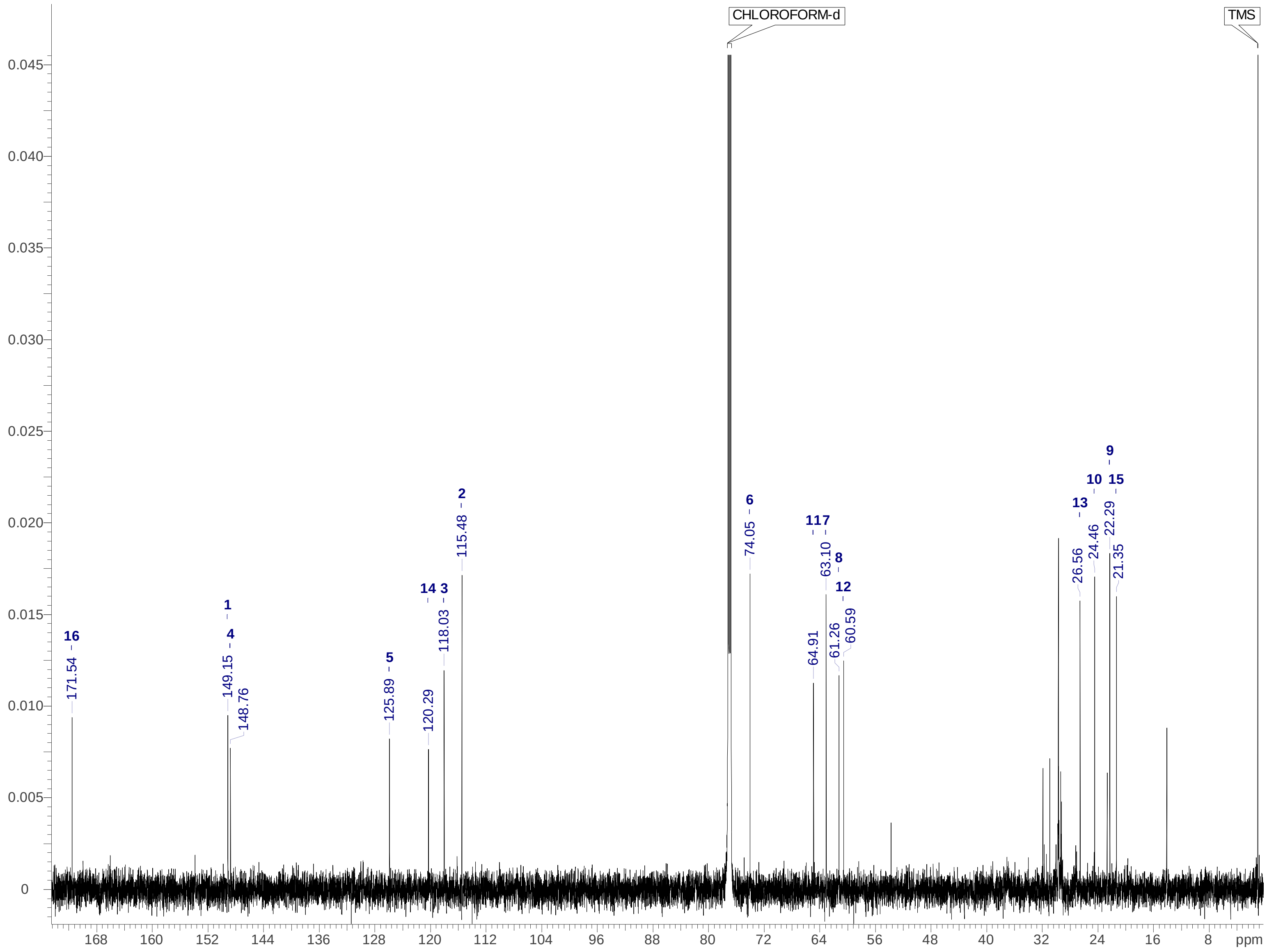

Figure S6. ^{13}C NMR spectrum (175 MHz) *ent*-clavilactone J in $CDCl_3$

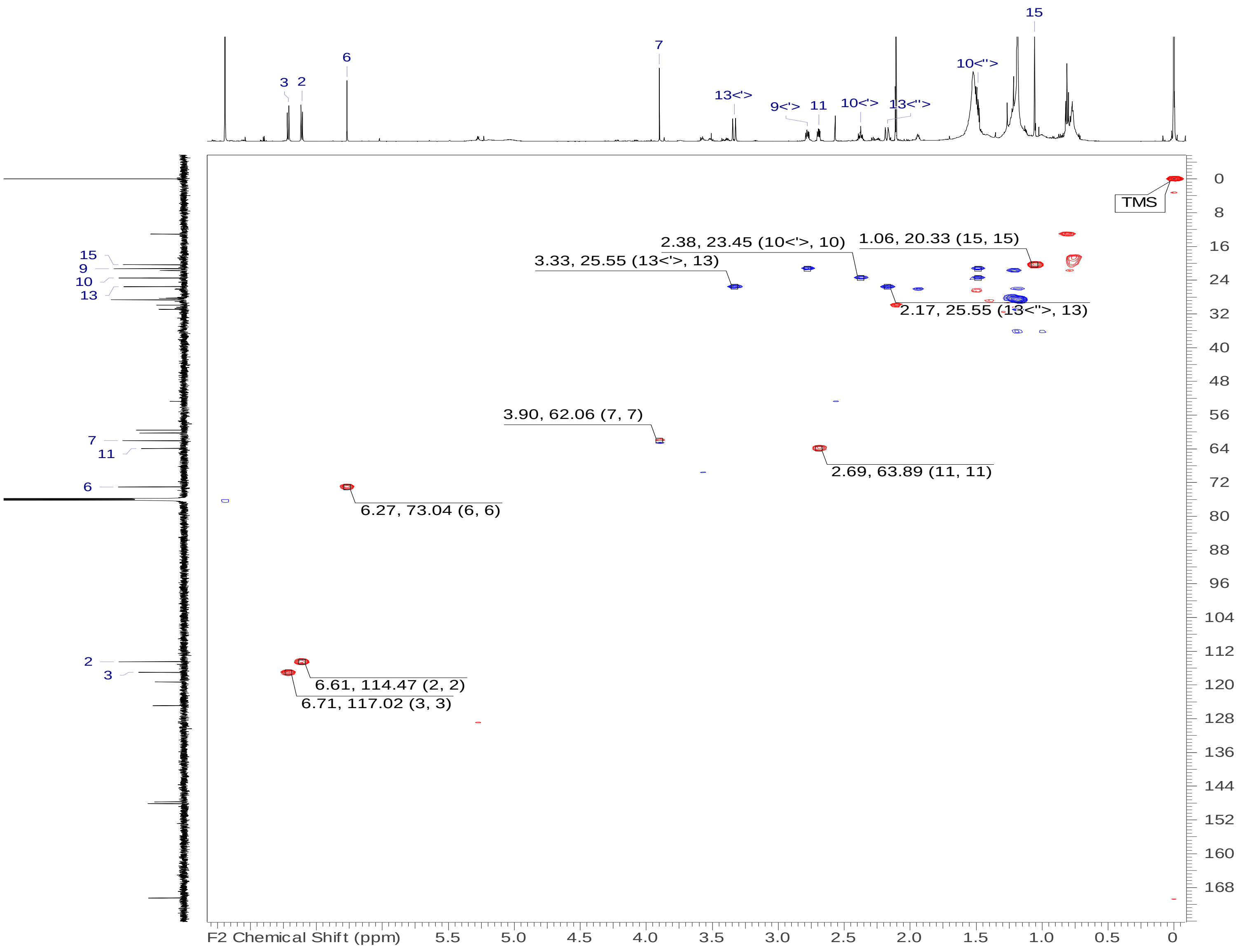

Figure S7. HSQC-DEPT NMR spectrum (700 MHz) *ent*-clavilactone J in $CDCl_3$

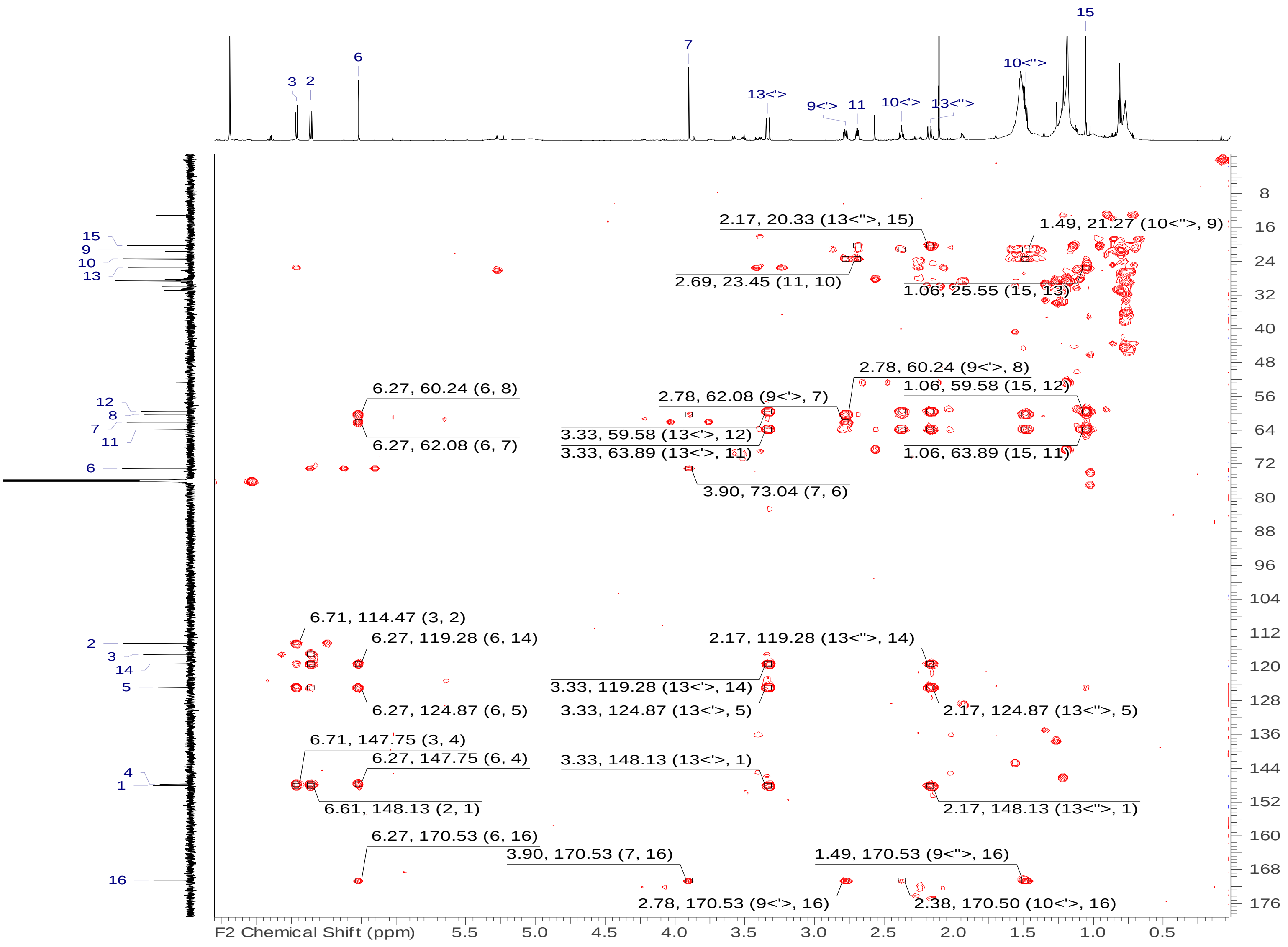

Figure S8. HMBC NMR spectrum (700 MHz) *ent*-clavilactone J in $CDCl_3$

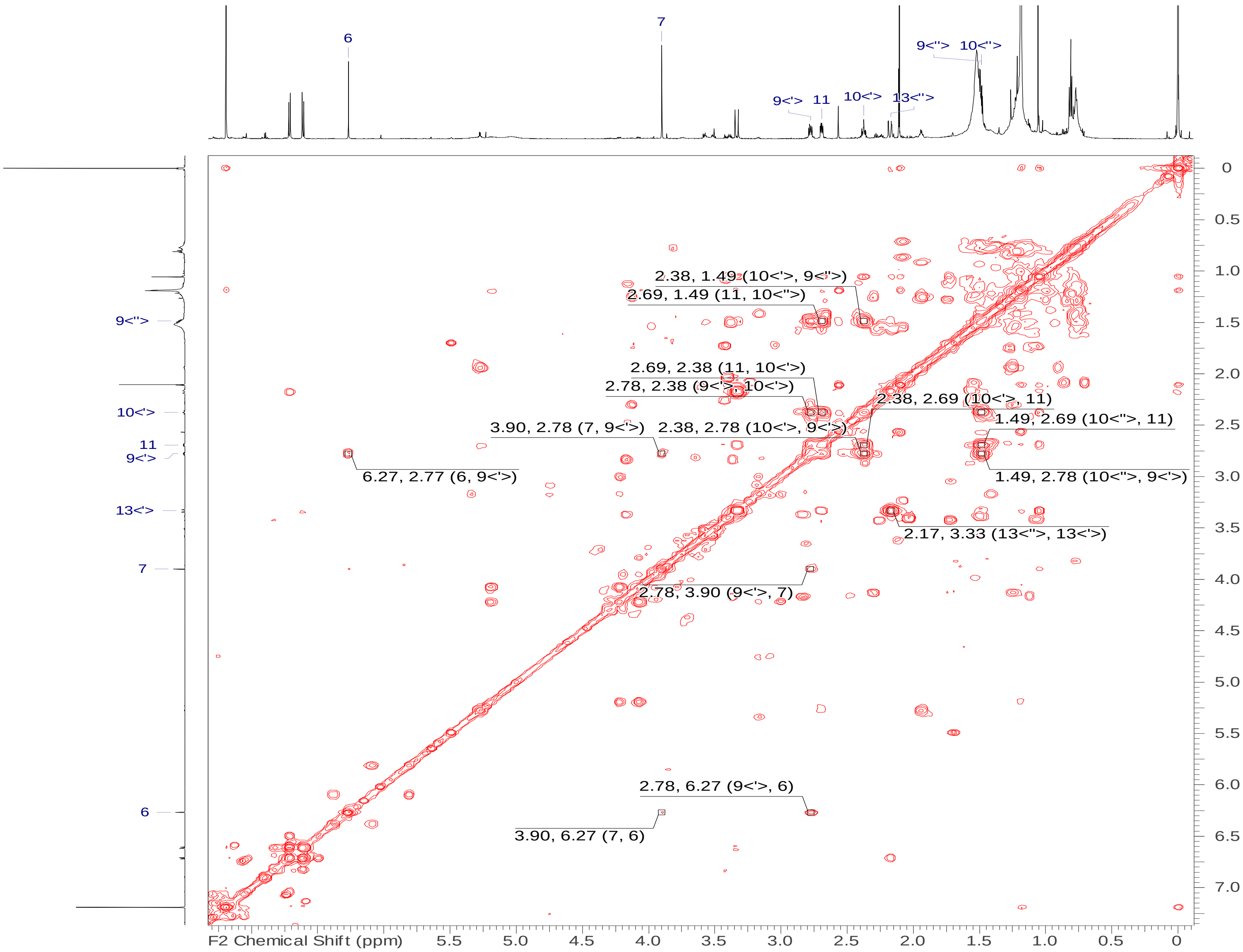

Figure S9. COSY NMR spectrum (700 MHz) *ent*-clavilactone J in $CDCl_3$

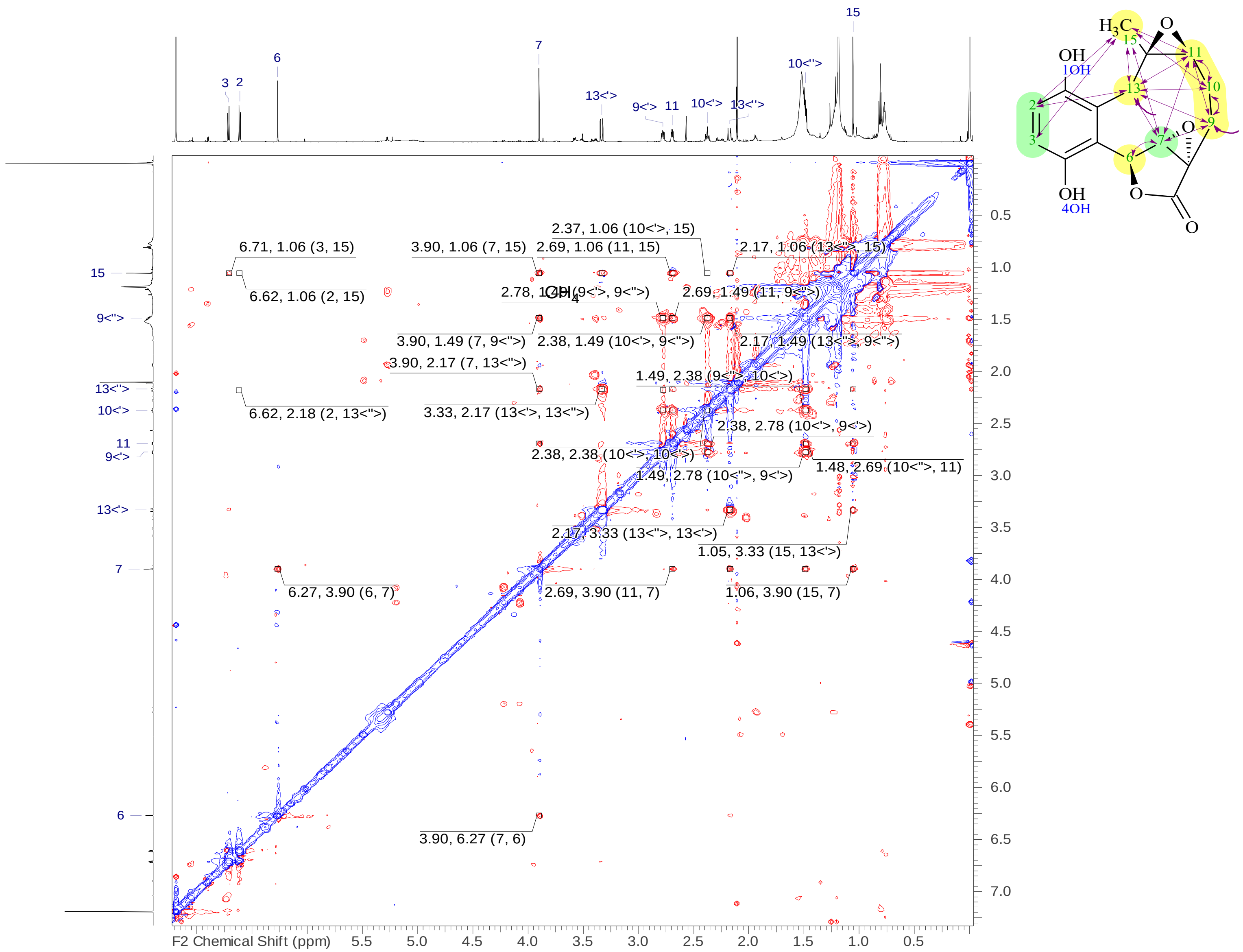

Figure S10. ROESY NMR spectrum (700 MHz) *ent*-clavilactone J in $CDCl_3$

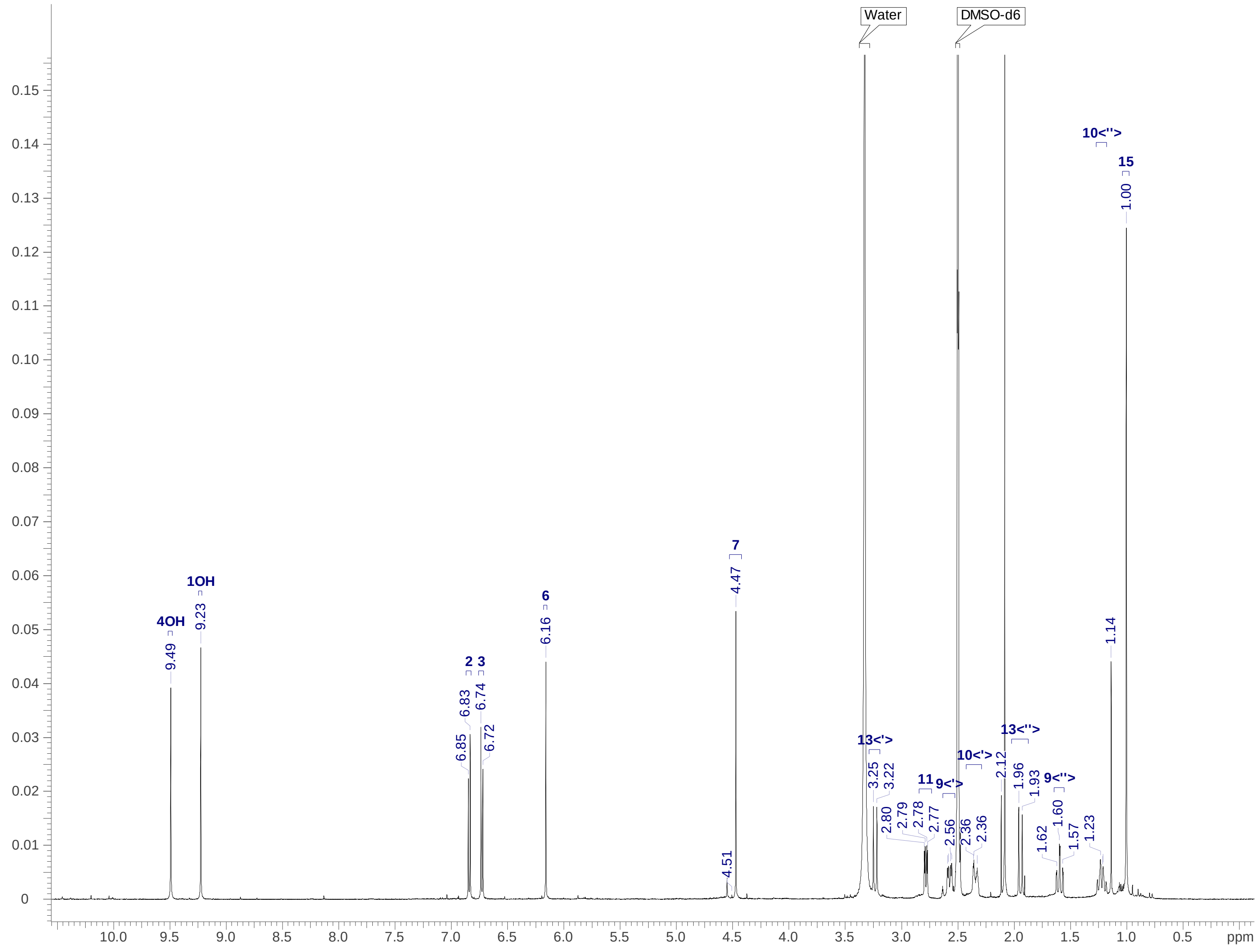

Figure S11. ^{1}H NMR spectrum (500 MHz) *ent*-clavilactone J in DMSO-D_6

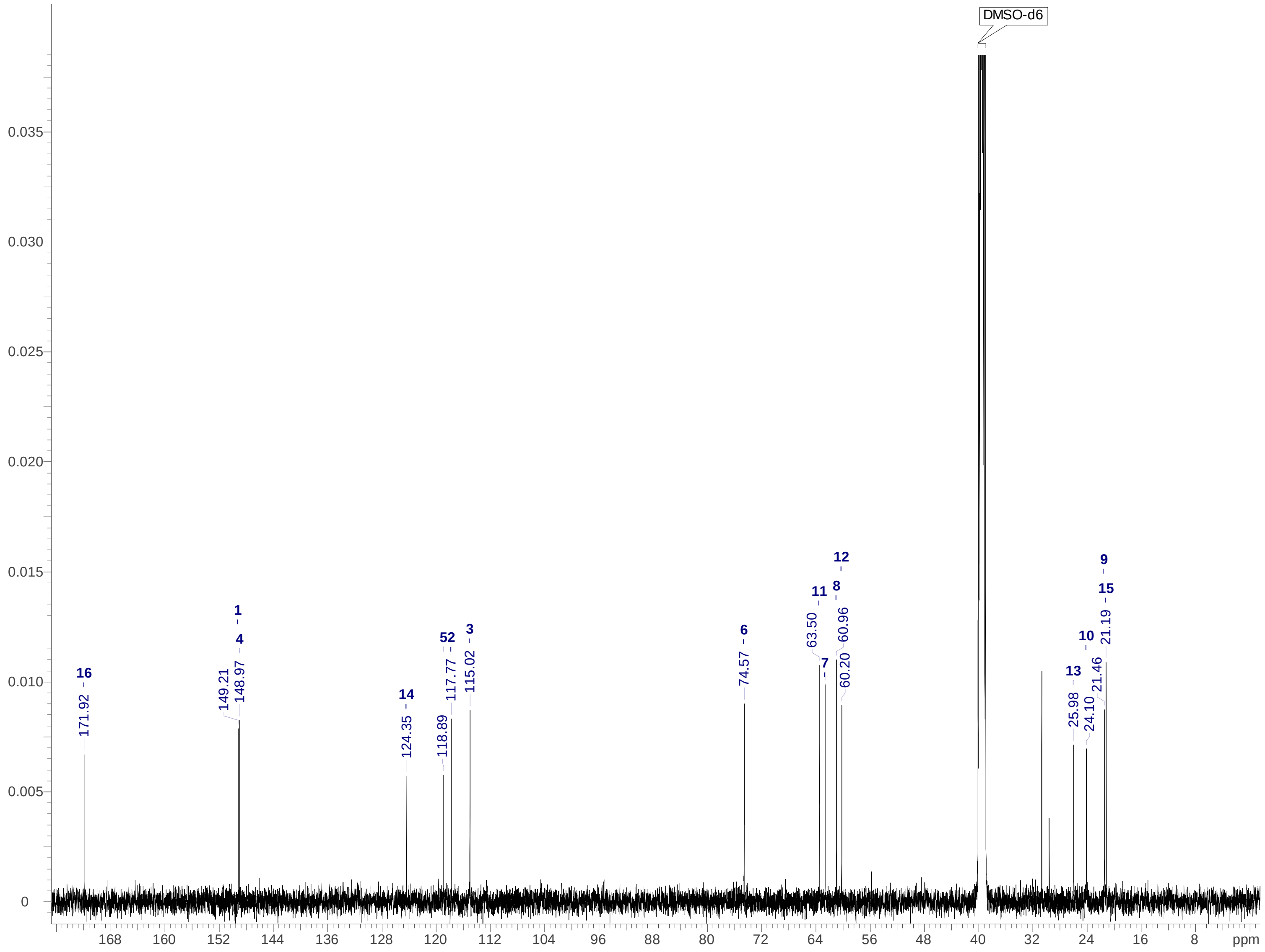

Figure S12. ^{13}C NMR spectrum (125 MHz) *ent*-clavilactone J in DMSO-D$_6$

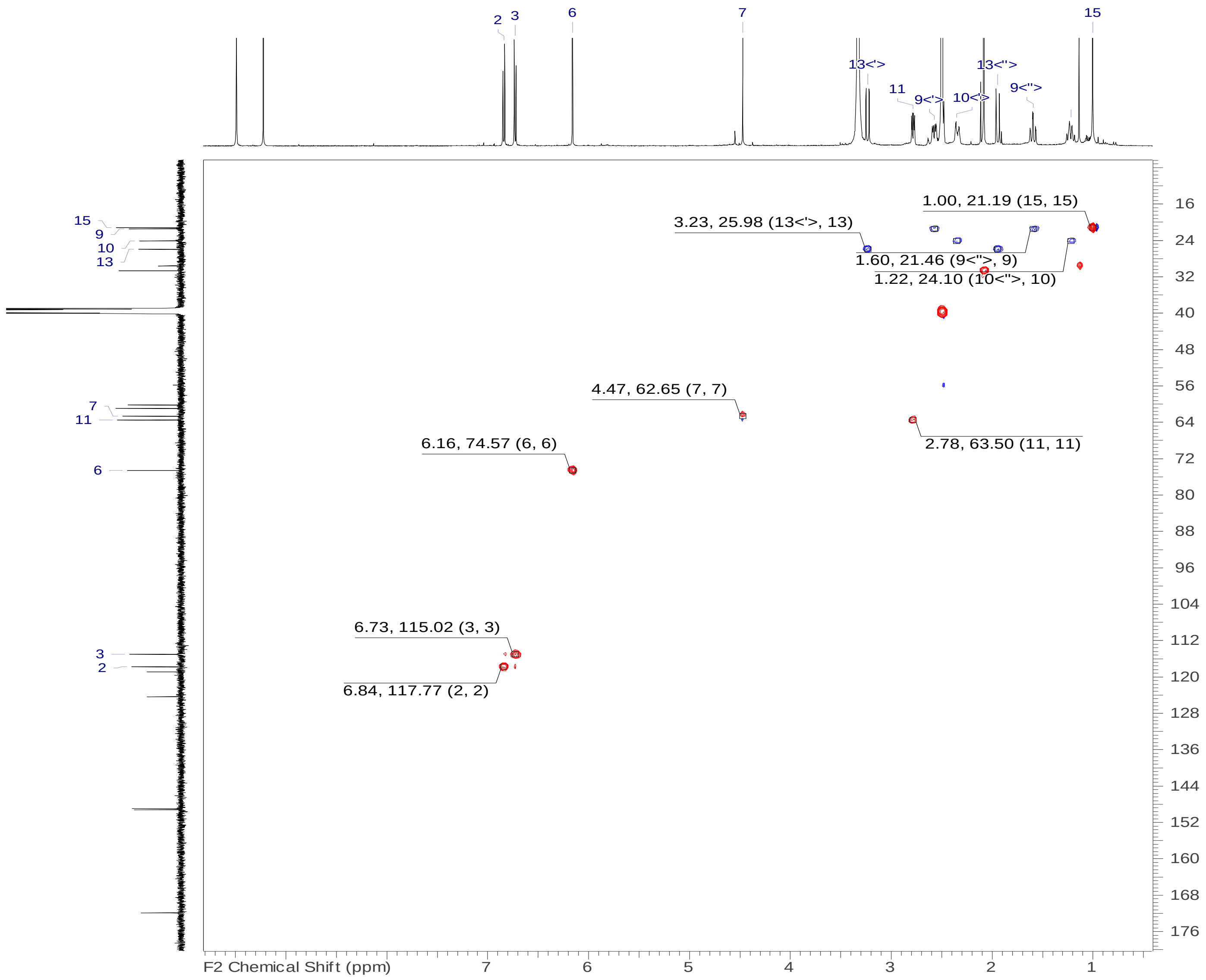

Figure S13. HSQC-DEPT NMR spectrum (500 MHz) *ent*-clavilactone J in DMSO-D_6

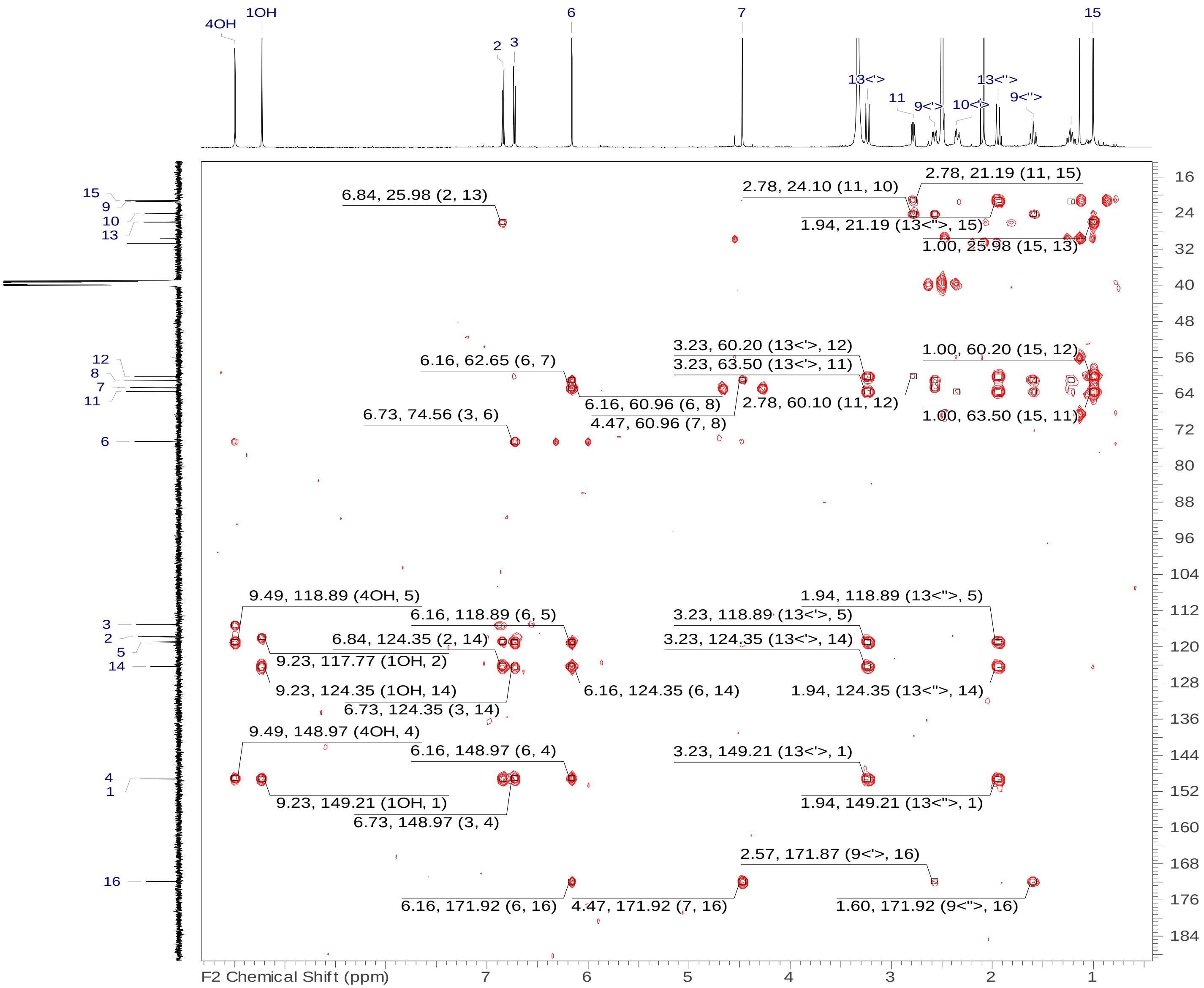

Figure S14. HMBC NMR spectrum (500 MHz) *ent*-clavilactone J in DMSO-D_6

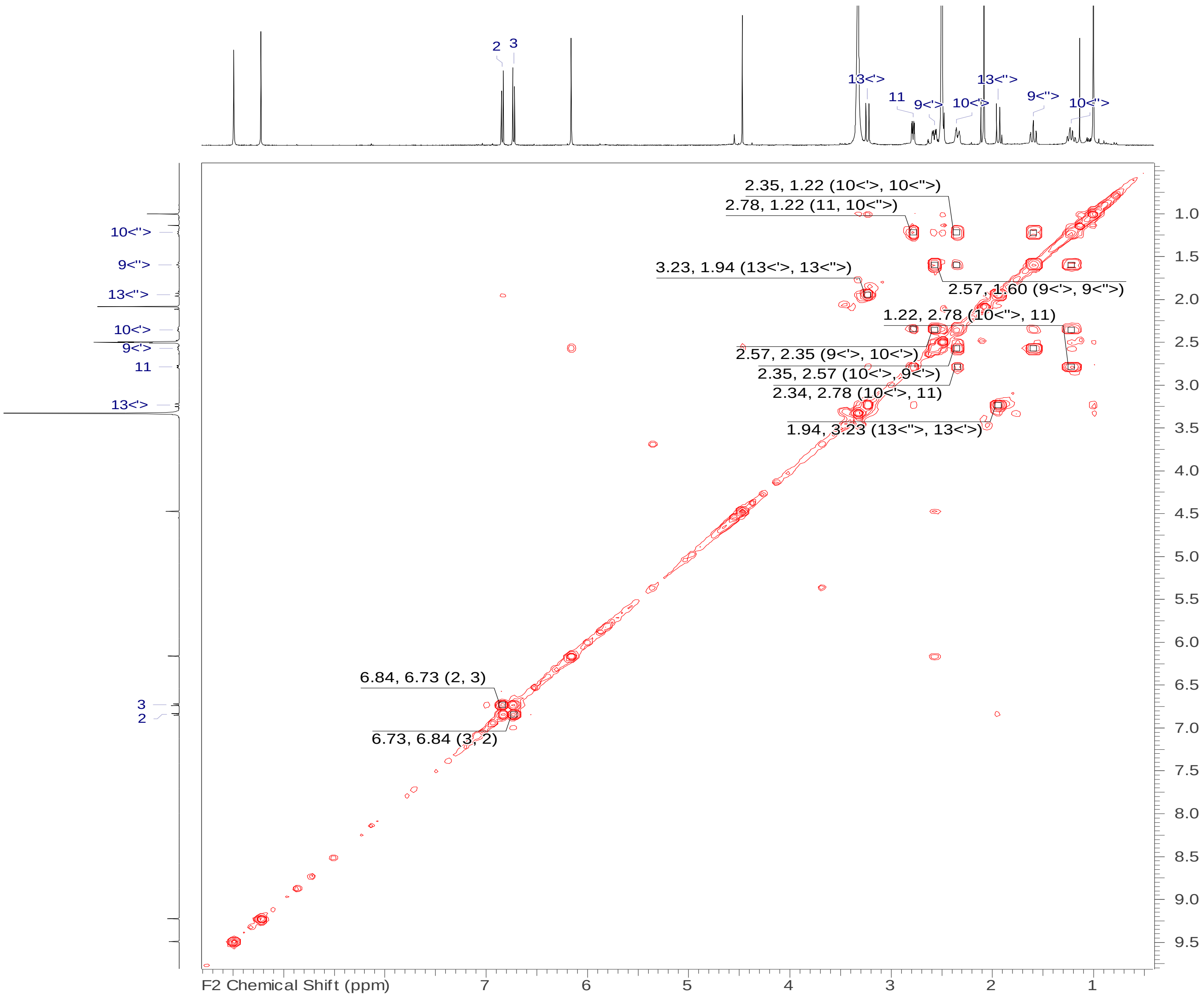

Figure S15. COSY NMR spectrum (500 MHz) *ent*-clavilactone J in DMSO-D_6

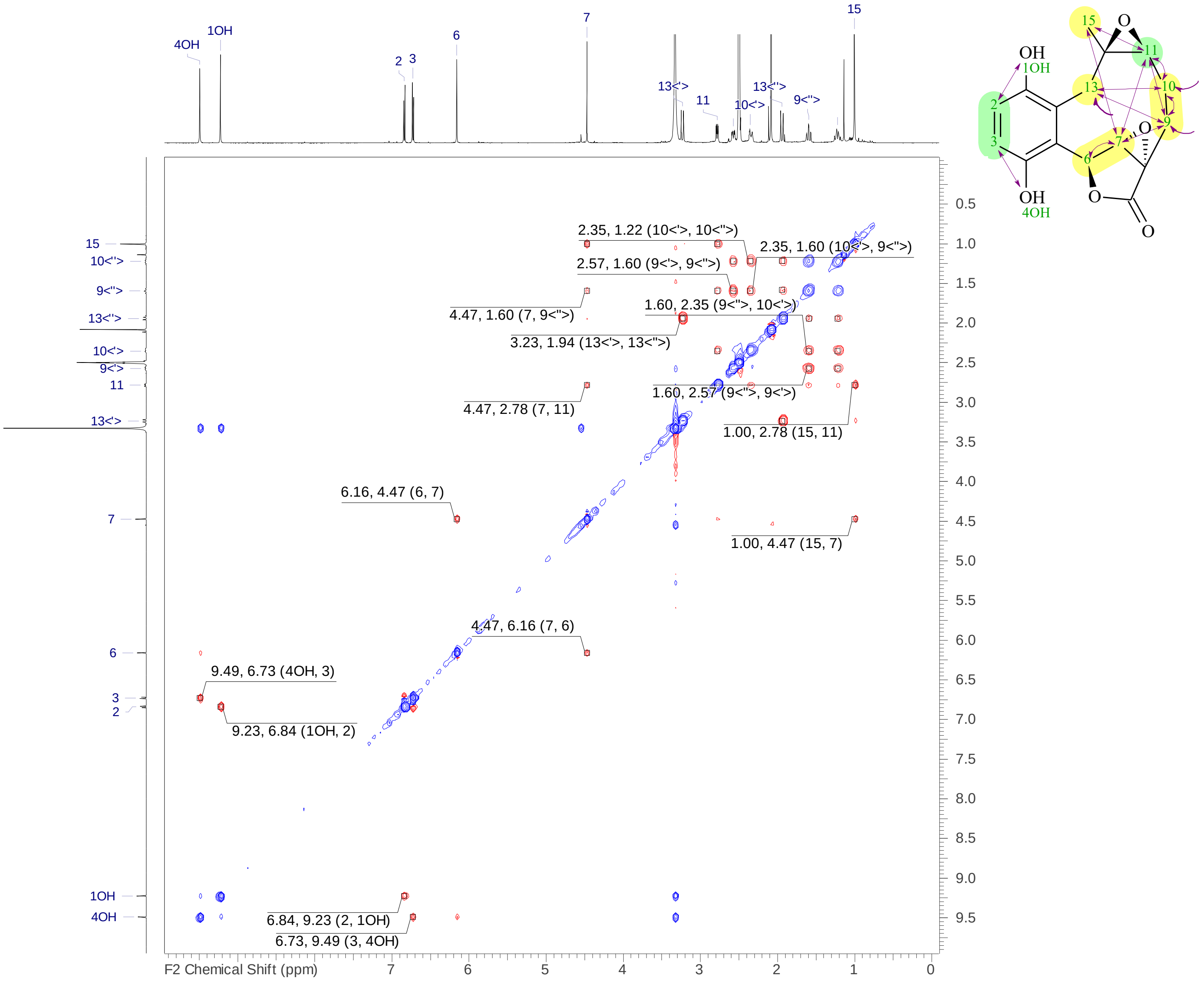

Figure S16. ROESY NMR spectrum (500 MHz) *ent*-clavilactone J in DMSO-D_6

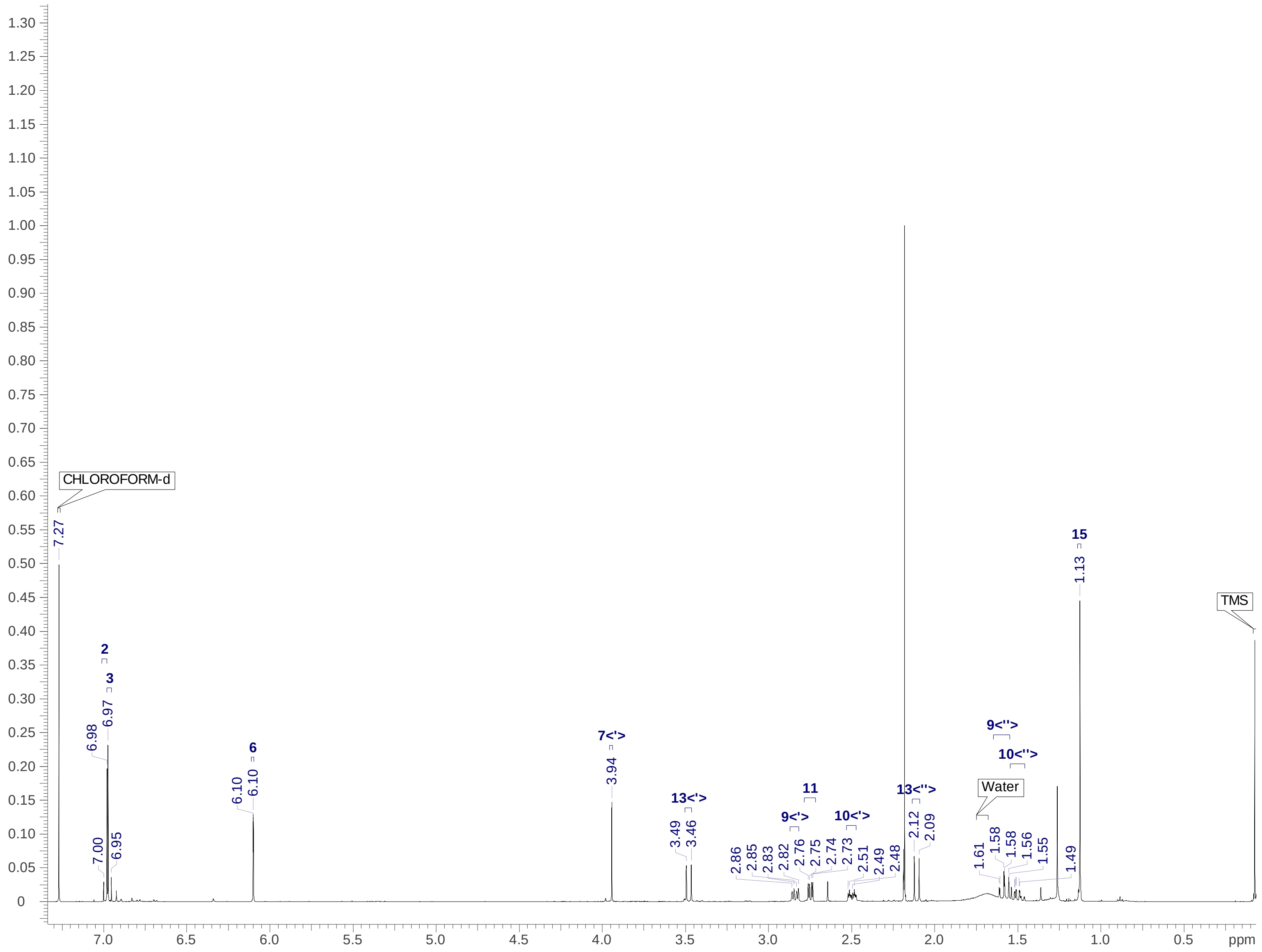

Figure S17. ^{1}H NMR spectrum (500 MHz) of *ent*-clavilactone J quinone in $CDCl_3$

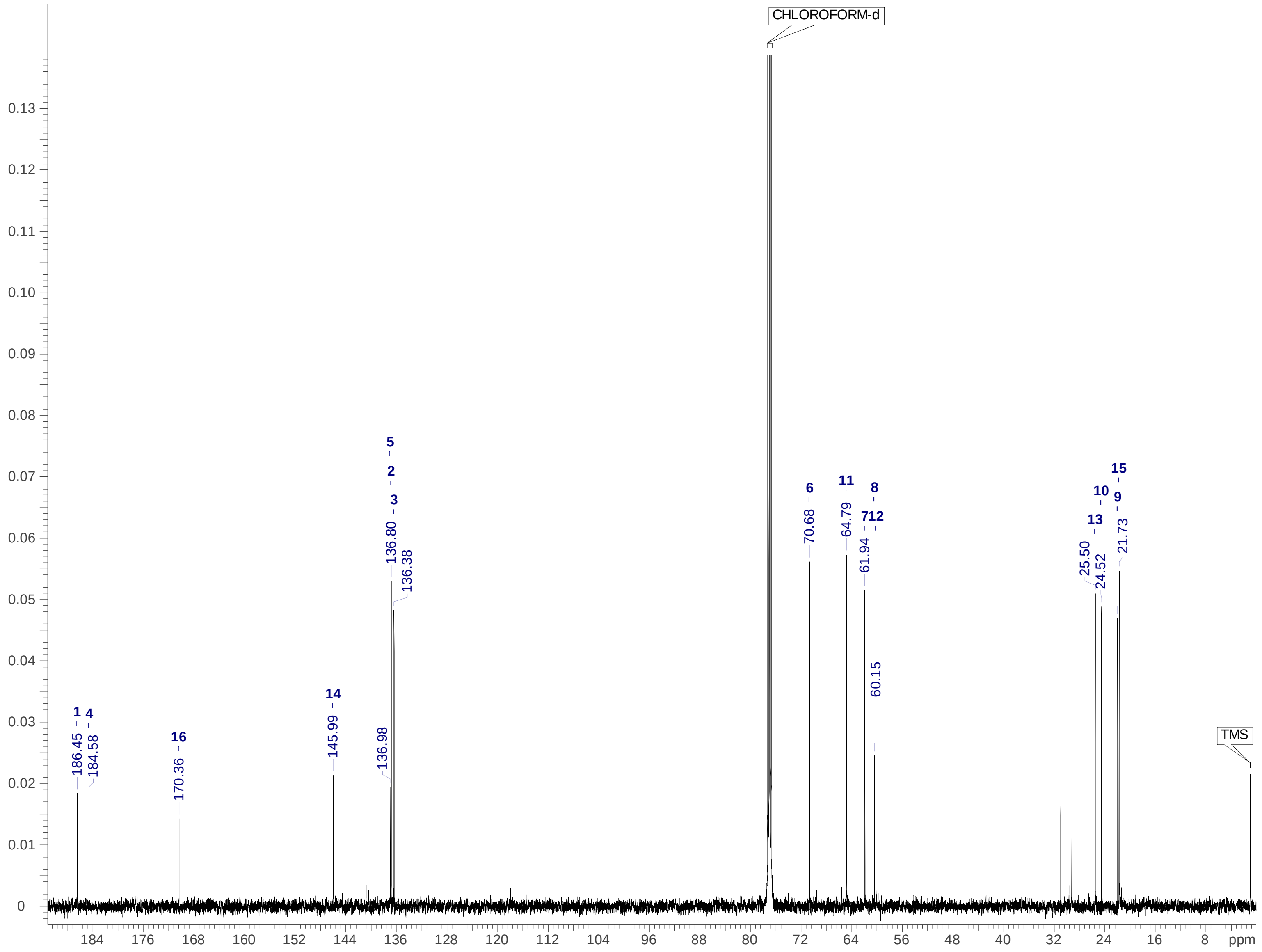

Figure S18. ^{13}C NMR spectrum (125 MHz) of *ent*-clavilactone J quinone in $CDCl_3$

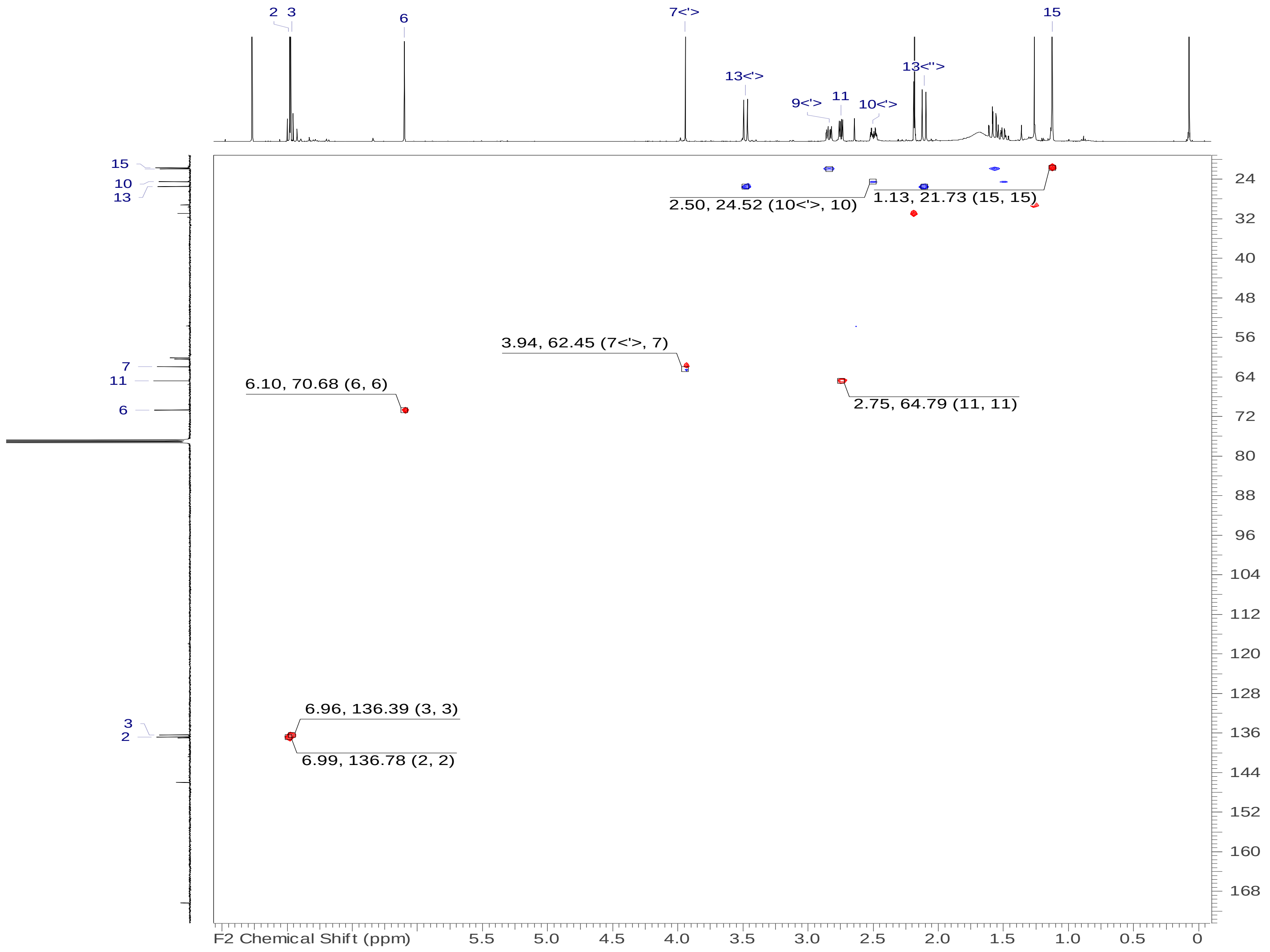

Figure S19. HSQC-DEPT NMR spectrum (500 MHz) *ent*-clavilactone J quinone in $CDCl_3$

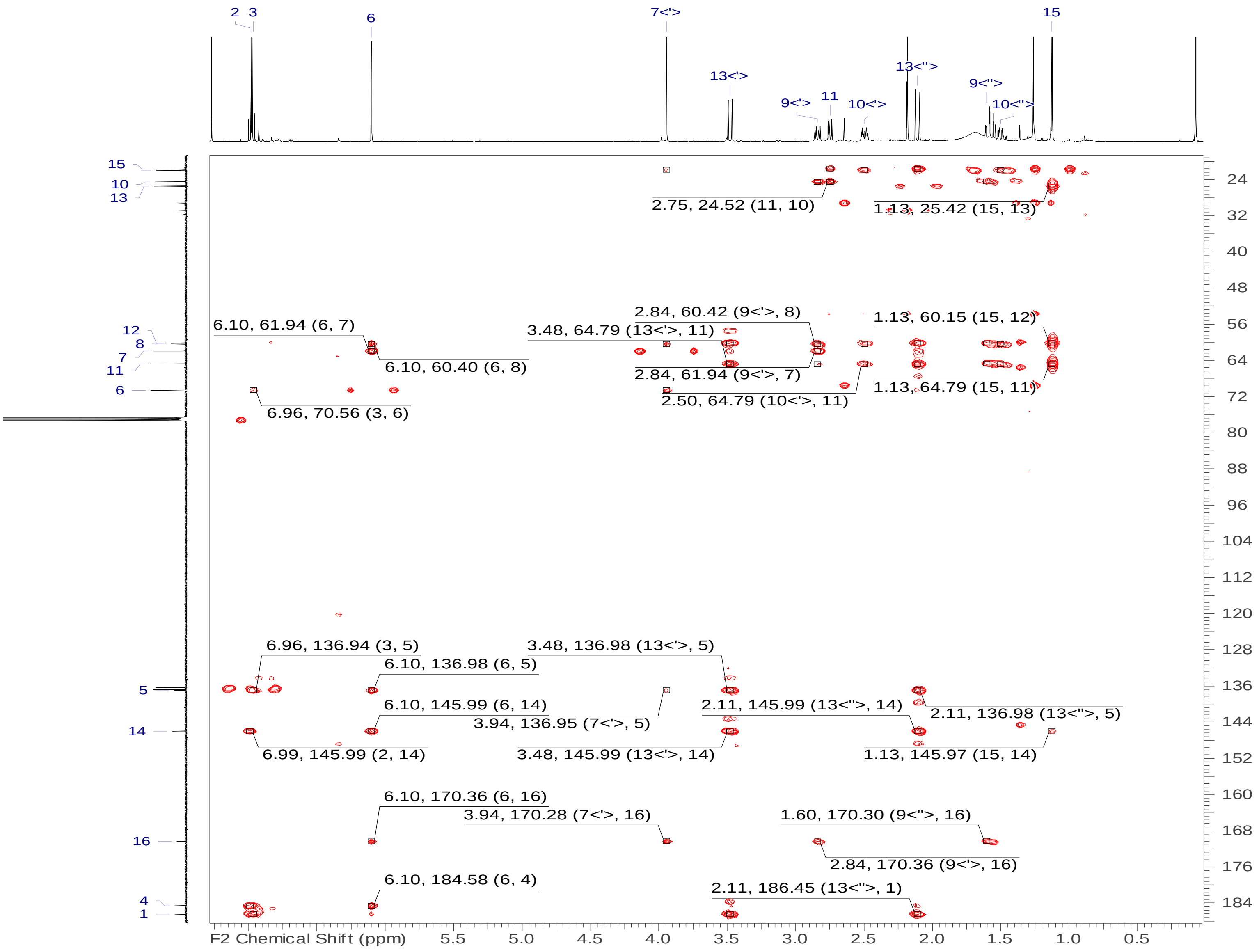

Figure S20. HMBC NMR spectrum (500 MHz) *ent*-clavilactone J quinone in $CDCl_3$

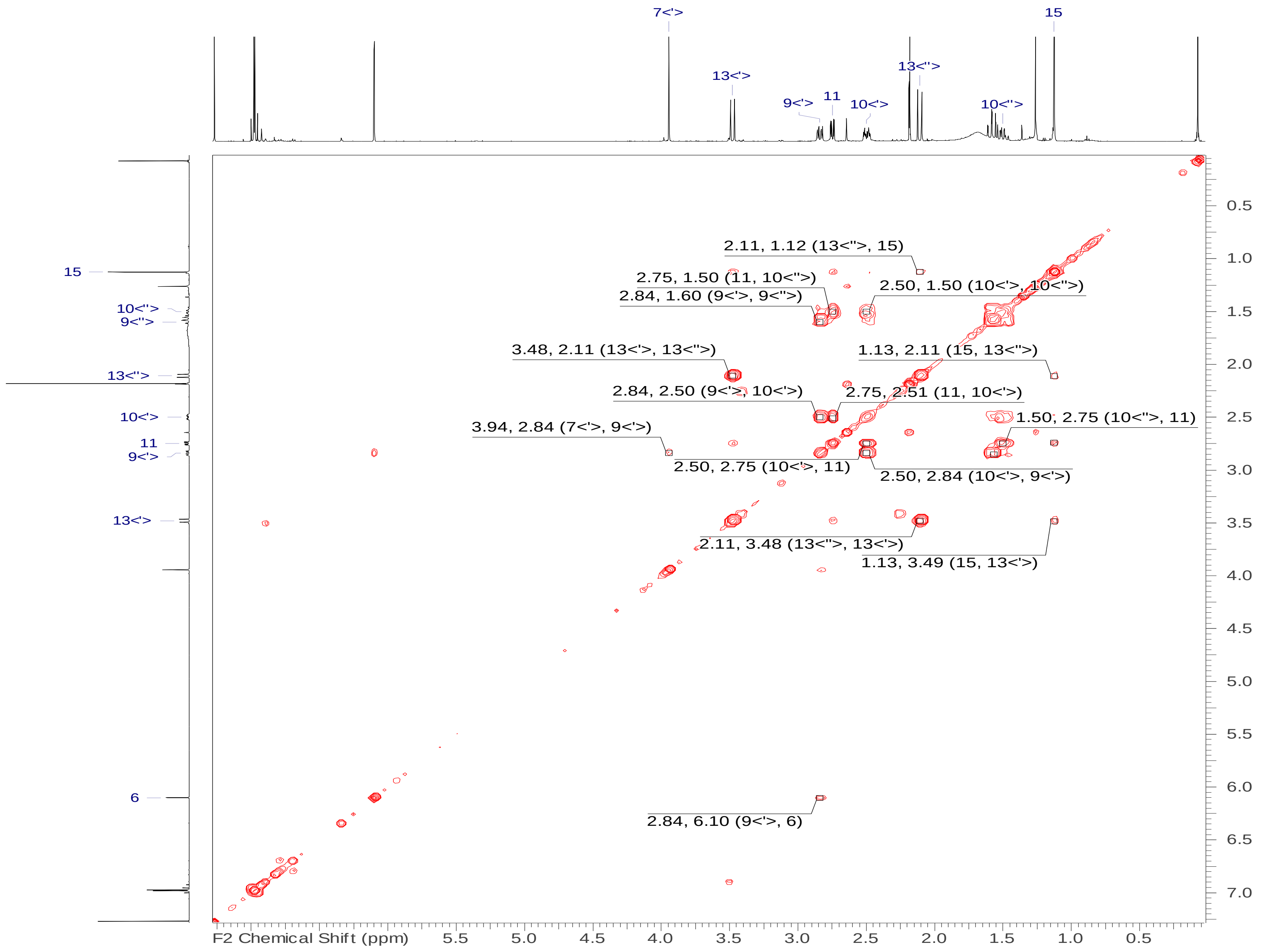

Figure S21. COSY NMR spectrum (500 MHz) *ent*-clavilactone J quinone in $CDCl_3$

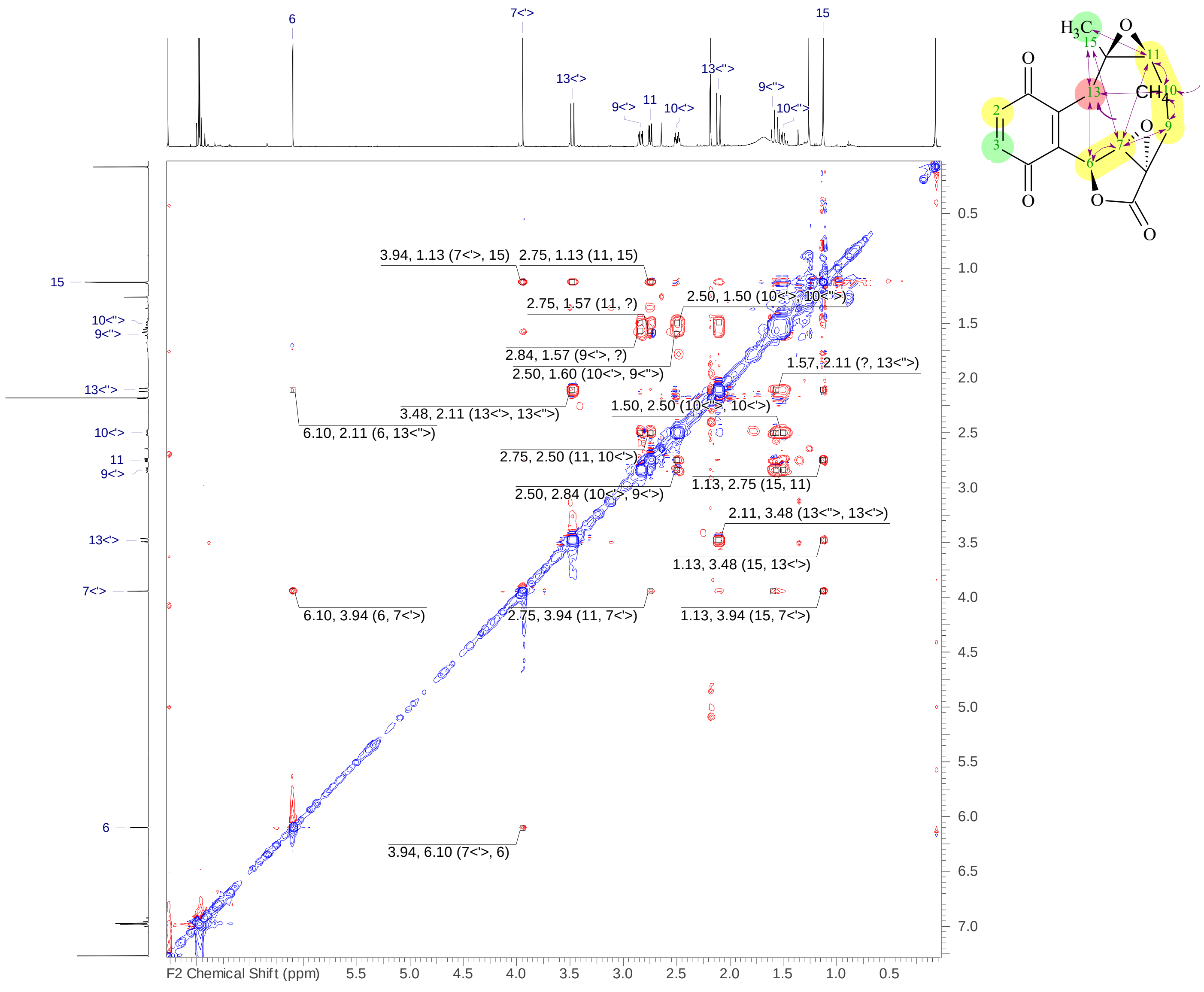

Figure S22. ROESY NMR spectrum (500 MHz) *ent*-clavilactone J quinone in $CDCl_3$

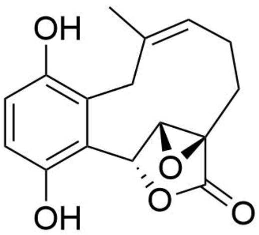

Clavilactone A

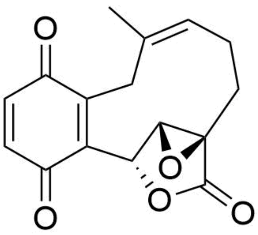

Clavilactone B

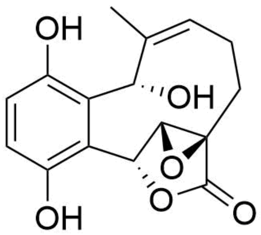

Clavilactone C

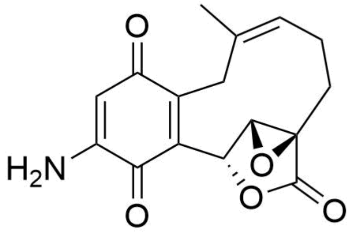

Clavilactone D

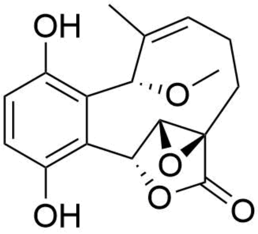

Clavilactone E

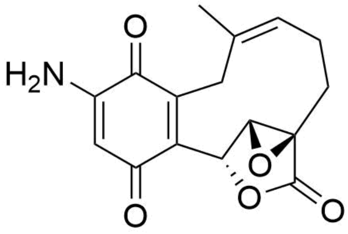

Clavilactone F

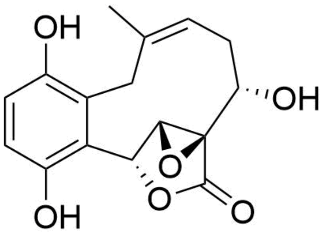

Clavilactone G

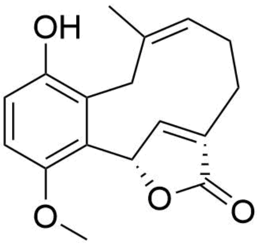

Clavilactone H

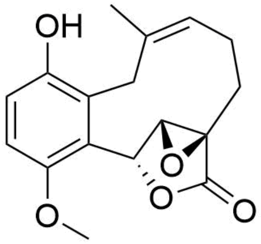

Clavilactone I

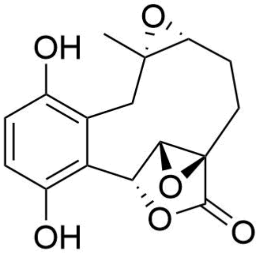

Clavilactone J

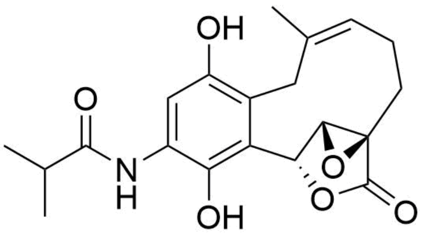

Clavilactone K

Figure S23. Overview of the clavilactones A – K.[1,2,3,4,5]

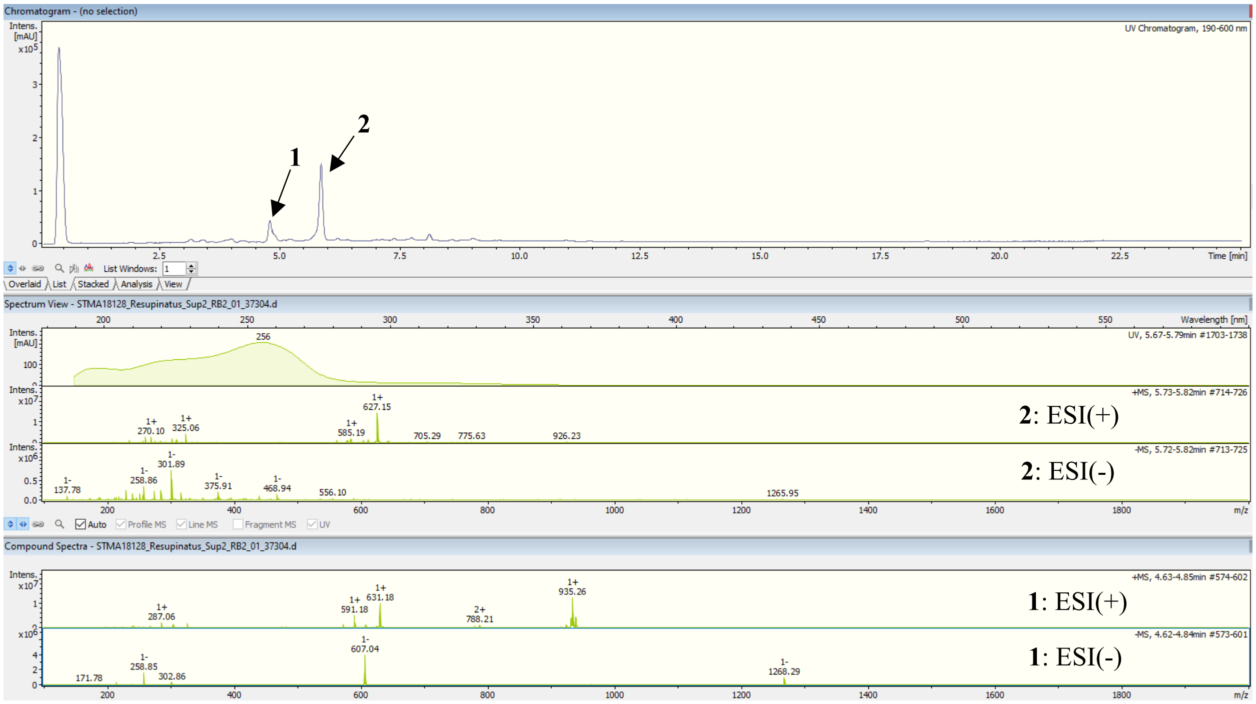

Figure S24. HPLC-UV/VIS-MS data of the supernatant crude extract obtained from *Resupinatus* sp. grown in MGP medium measured with positive and negative modes, respectively. Data extracted for main metabolites *ent*-clavilactone J (**1**) and *ent*-clavilactone J quinone (**2**).

References for Supporting Informantion

(1) Takao, K. I.; Mori, K.; Kasuga, K.; Nanamiya, R.; Namba, A.; Fukushima, Y.; Nemoto, R.; Mogi, T.; Yasui, H.; Ogura, A.; Yoshida, K.; Tadano, K. I. Total synthesis of clavilactones. *J. Org. Chem.* **2018**, *83* (13), 7060–7075. https://doi.org/10.1021/acs.joc.7b03268.

(2) Sun, Z.; Zhu, N.; Zhou, M.; Huo, X.; Wu, H.; Tian, Y.; Yang, J.; Ma, G.; Yang, Y. L.; Xu, X. Clavipines A-C, Antiproliferative meroterpenoids with a fused azepine skeleton from the basidiomycete *Clitocybe clavipes*. *Org. Chem. Front.* **2019**, *6* (22), 3759–3765. https://doi.org/10.1039/c9qo01005j.

(3) Zhaocui, S.; Xudong, X.; Hanqiao, L.; Xinyi, X.; Guoxu, M.; Leiling, S. Five new meroterpenoids from the fruiting bodies of the basidiomycete *Clitocybe clavipes* with cytotoxic activity. *Molecules* **2019**, *24* (22), 1–10. https://doi.org/10.3390/molecules24224015.

(4) Hou, Y.; Li, Q.; Chen, M.; Wu, H.; Yang, J.; Sun, Z.; Xu, X.; Ma, G. Novel geranylhydroquinone derived meroterpenoids from the fungus *Clitocybe clavipes* and their cytotoxic activity. *Fitoterapia* **2022**, *161*, 105251. https://doi.org/10.1016/j.fitote.2022.105251.

(5) Novitskiy, I. M.; Kutateladze, A. G. Brief overview of recently reported misassigned natural products and their in silico revisions enabled by DU8ML, a machine learning-augmented DFT computational NMR method. *Nat. Prod. Rep.* **2022**, *39*, 2003-2007 https://doi.org/10.1039/d2np00051b.
