# Supplementary figures and images for: ent-Clavilactone J and Its Quinone Derivative, Meroterpenoids from the Fungus Resupinatus sp
Source: J Nat Prod. 2023 Nov 6;86(11):2580–4. doi: 10.1021/acs.jnatprod.3c00174 (PMC10683060; doi:10.1021/acs.jnatprod.3c00174)

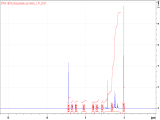

Supplement: Supplementary file 2 — np3c00174_si_002.zip [file np3c00174_si_002.zip › ent-clavilactone J in CDCl3 kha19_24660Sm/1 1H/pdata/1/thumb.png]

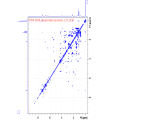

Supplement: Supplementary file 2 — np3c00174_si_002.zip [file np3c00174_si_002.zip › ent-clavilactone J in CDCl3 kha19_24660Sm/2 COSY/pdata/1/thumb.png]

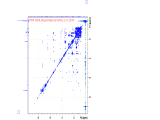

Supplement: Supplementary file 2 — np3c00174_si_002.zip [file np3c00174_si_002.zip › ent-clavilactone J in CDCl3 kha19_24660Sm/3 TOCSY/pdata/1/thumb.png]

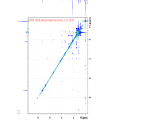

Supplement: Supplementary file 2 — np3c00174_si_002.zip [file np3c00174_si_002.zip › ent-clavilactone J in CDCl3 kha19_24660Sm/4 ROESY/pdata/1/thumb.png]

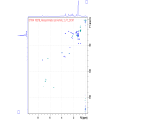

Supplement: Supplementary file 2 — np3c00174_si_002.zip [file np3c00174_si_002.zip › ent-clavilactone J in CDCl3 kha19_24660Sm/5 HSQC-DEPT/pdata/1/thumb.png]

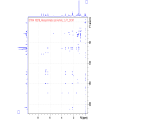

Supplement: Supplementary file 2 — np3c00174_si_002.zip [file np3c00174_si_002.zip › ent-clavilactone J in CDCl3 kha19_24660Sm/6 HMBC/pdata/1/thumb.png]
