# Supplementary material for: ent-Clavilactone J and Its Quinone Derivative, Meroterpenoids from the Fungus Resupinatus sp
Source: J Nat Prod. 2023 Nov 6;86(11):2580–4. doi: 10.1021/acs.jnatprod.3c00174 (PMC10683060; doi:10.1021/acs.jnatprod.3c00174)
Supplement: Supplementary file 2 — np3c00174_si_002.zip [file np3c00174_si_002.zip › ent-clavilactone J in DMSO ppa19_21978Fa/3 1H/pdata/1/email_ppa19_21978Fa_3_1.pdf]

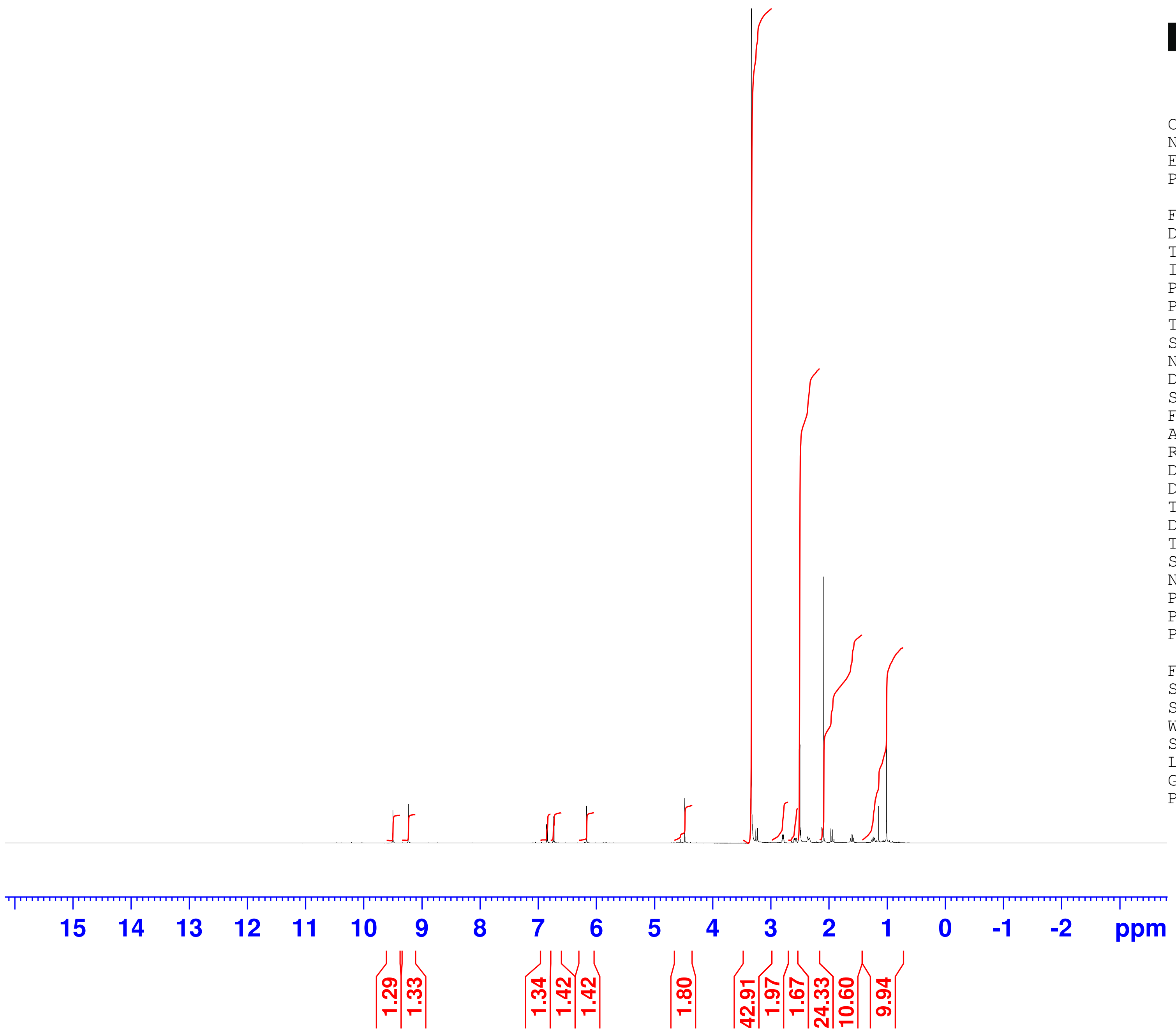

Current Data Parameters
NAME ppa19_21978Fa
EXPNO 3
PROCNO 1

F2 - Acquisition Parameters
Date_ 20210718
Time 14.33 h
INSTRUM spect
PROBHD Z113652_0281 (
PULPROG zg30
TD 65536
SOLVENT DMSO
NS 24
DS 2
SWH 10000.000 Hz
FIDRES 0.305176 Hz
AQ 3.2767999 sec
RG 184.28
DW 50.000 usec
DE 14.07 usec
TE 298.0 K
D1 1.00000000 sec
TD0 1
SFO1 500.2830892 MHz
NUC1 1H
P0 2.99 usec
P1 8.96 usec
PLW1 25.00000000 W

F2 - Processing parameters
SI 65536
SF 500.2800000 MHz
WDW EM
SSB 0
LB 0.30 Hz
GB 0
PC 1.00
