# Supplementary material for: ent-Clavilactone J and Its Quinone Derivative, Meroterpenoids from the Fungus Resupinatus sp
Source: J Nat Prod. 2023 Nov 6;86(11):2580–4. doi: 10.1021/acs.jnatprod.3c00174 (PMC10683060; doi:10.1021/acs.jnatprod.3c00174)
Supplement: Supplementary file 2 — np3c00174_si_002.zip [file np3c00174_si_002.zip › ent-clavilactone J in DMSO ppa19_21978Fa/4 ROESY/pdata/1/email_ppa19_21978Fa_4_1.pdf]

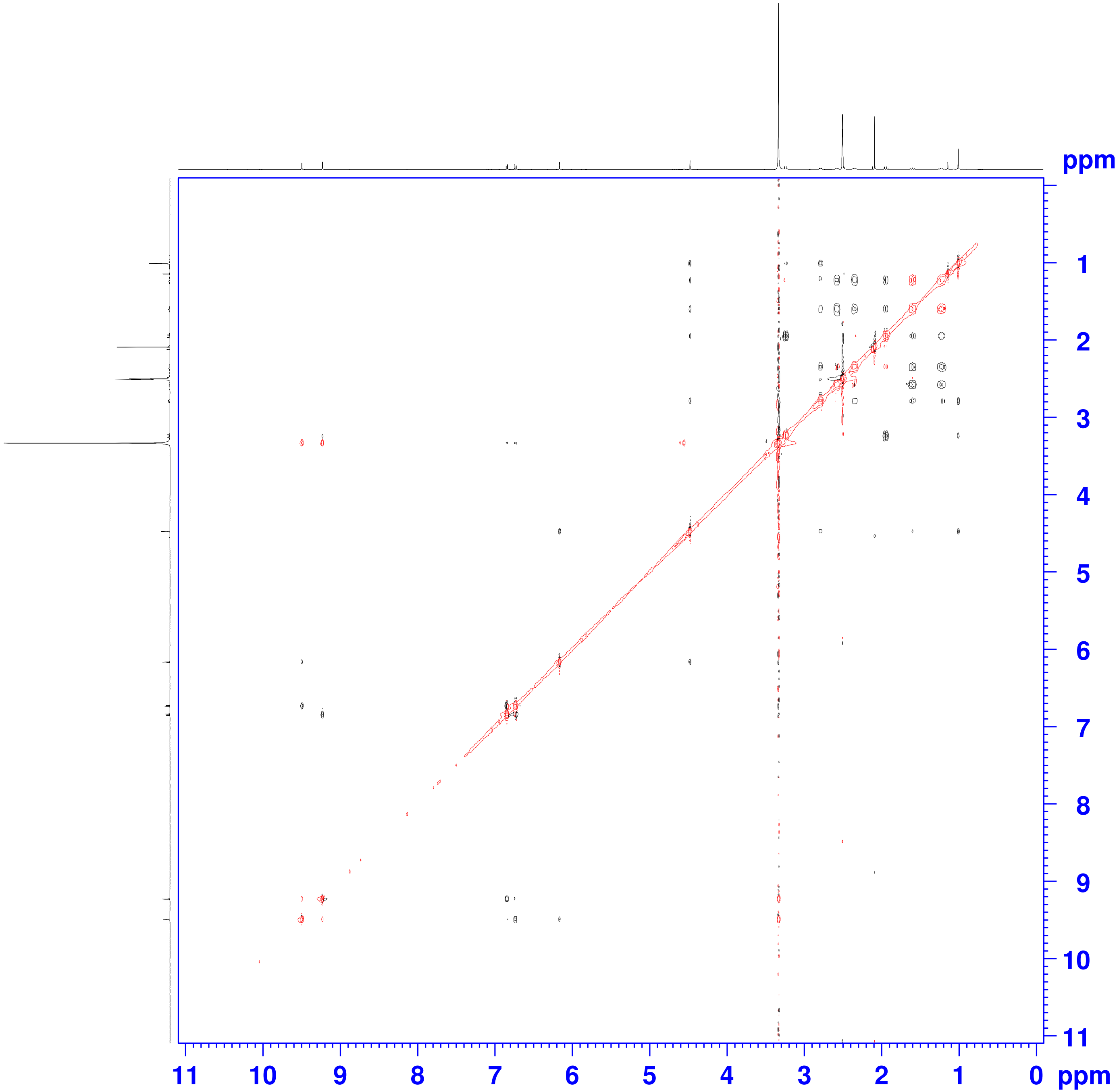

BRUKER

Current Data Parameters

NAME	ppa19_21978Fa	
EXPNO	4	
PROCNO	1	

F2 - Acquisition Parameters

Date_	20210718	
Time	14.36	h
INSTRUM	spect	
PROBHD	Z113652_0281 (	
PULPROG	roesyphpp.2	
TD	2048	
SOLVENT	DMSO	
NS	16	
DS	32	
SWH	5597.015	Hz
FIDRES	5.465835	Hz
AQ	0.1829547	sec
RG	1820	
DW	89.333	usec
DE	6.50	usec
TE	298.0	K
D0	0.00007960	sec
D1	1.50000000	sec
D11	0.03000000	sec
D12	0.00002000	sec
IN0	0.00017860	sec
L4	1157	
P15	500000.00	usec
TDav	1	
SFO1	500.2827515	MHz
NUC1	1H	
P1	8.96	usec
P17	2500.00	usec
P25	216.00	usec
PLW1	25.00300026	W
PLW10	2.23040009	W
PLW27	0.17209999	W

F1 - Acquisition parameters

TD	256	
SFO1	500.2828	MHz
FIDRES	43.743000	Hz
SW	11.192	ppm
FnMODE	States-TPPI	

F2 - Processing parameters

SI	1024	
SF	500.2800000	MHz
WDW	QSINE	
SSB	2	
LB	0	Hz
GB	0	
PC	1.00	

F1 - Processing parameters

SI	1024	
MC2	States-TPPI	
SF	500.2800000	MHz
WDW	QSINE	
SSB	2	
LB	0	Hz
GB	0	
