# Supplementary material for: ent-Clavilactone J and Its Quinone Derivative, Meroterpenoids from the Fungus Resupinatus sp
Source: J Nat Prod. 2023 Nov 6;86(11):2580–4. doi: 10.1021/acs.jnatprod.3c00174 (PMC10683060; doi:10.1021/acs.jnatprod.3c00174)
Supplement: Supplementary file 2 — np3c00174_si_002.zip [file np3c00174_si_002.zip › ent-clavilactone J in DMSO ppa19_21978Fa/5 HMBC/pdata/1/email_ppa19_21978Fa_5_1.pdf]

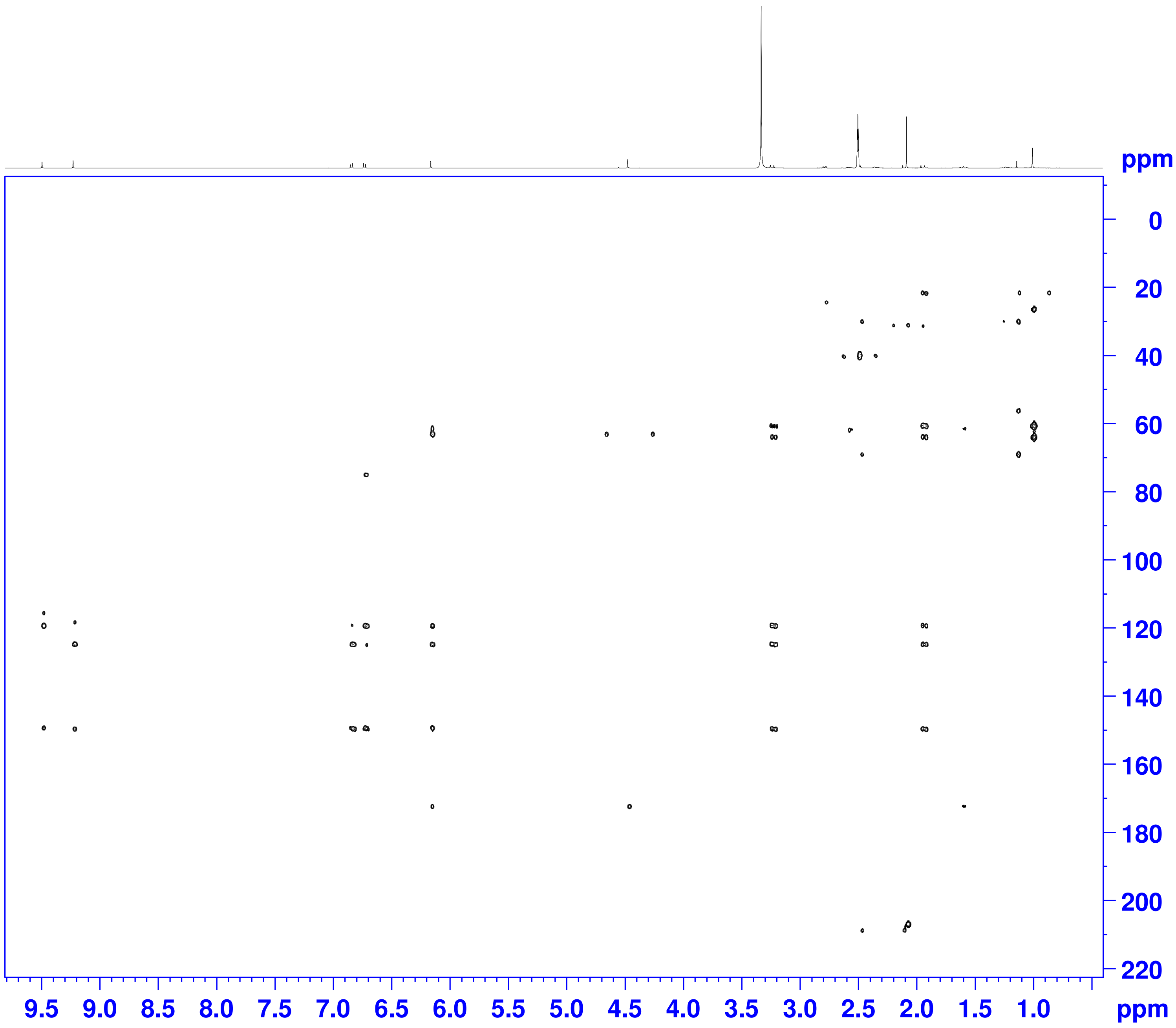

Current Data Parameters

NAME	ppa19_21978Fa	
EXPNO	5	
PROCNO	1	

F2 - Acquisition Parameters

Date_	20210718	
Time	17.11	h
INSTRUM	spect	
PROBHD	Z113652_0281 (	
PULPROG	hmbcgplpndqf	
TD	2048	
SOLVENT	DMSO	
NS	48	
DS	16	
SWH	4708.098	Hz
FIDRES	4.597752	Hz
AQ	0.2174976	sec
RG	2050	
DW	106.200	usec
DE	6.50	usec
TE	298.0	K
CNST2	145.0000000	
CNST13	6.0000000	
D0	0.00000300	sec
D1	1.50000000	sec
D2	0.00344828	sec
D6	0.08333334	sec
D16	0.00020000	sec
IN0	0.00001690	sec
TDav	1	
SFO1	500.2825557	MHz
NUC1	1H	
P1	8.96	usec
P2	17.92	usec
PLW1	25.00300026	W
SFO2	125.8087146	MHz
NUC2	13C	
P3	9.00	usec
PLW2	86.00000000	W
GPNAM[1]	SMSQ10.100	
GPZ1	50.00	%
GPNAM[2]	SMSQ10.100	
GPZ2	30.00	%
GPNAM[3]	SMSQ10.100	
GPZ3	40.10	%
P16	1000.00	usec

F1 - Acquisition parameters

TD	256	
SFO1	125.8087	MHz
FIDRES	231.139053	Hz
SW	235.165	ppm
FnMODE	QF	

F2 - Processing parameters

SI	2048	
SF	500.2800000	MHz
WDW	SINE	
SSB	0	
LB	0	Hz
GB	0	
PC	1.40	

F1 - Processing parameters

SI	1024	
MC2	QF	
SF	125.7955061	MHz
WDW	SINE	
SSB	0	
LB	0	Hz
GB	0	
