# Supplementary material for: ent-Clavilactone J and Its Quinone Derivative, Meroterpenoids from the Fungus Resupinatus sp
Source: J Nat Prod. 2023 Nov 6;86(11):2580–4. doi: 10.1021/acs.jnatprod.3c00174 (PMC10683060; doi:10.1021/acs.jnatprod.3c00174)
Supplement: Supplementary file 2 — np3c00174_si_002.zip [file np3c00174_si_002.zip › ent-clavilactone J in DMSO ppa19_21978Fa/6 HSQC-DEPT/pdata/1/email_ppa19_21978Fa_6_1.pdf]

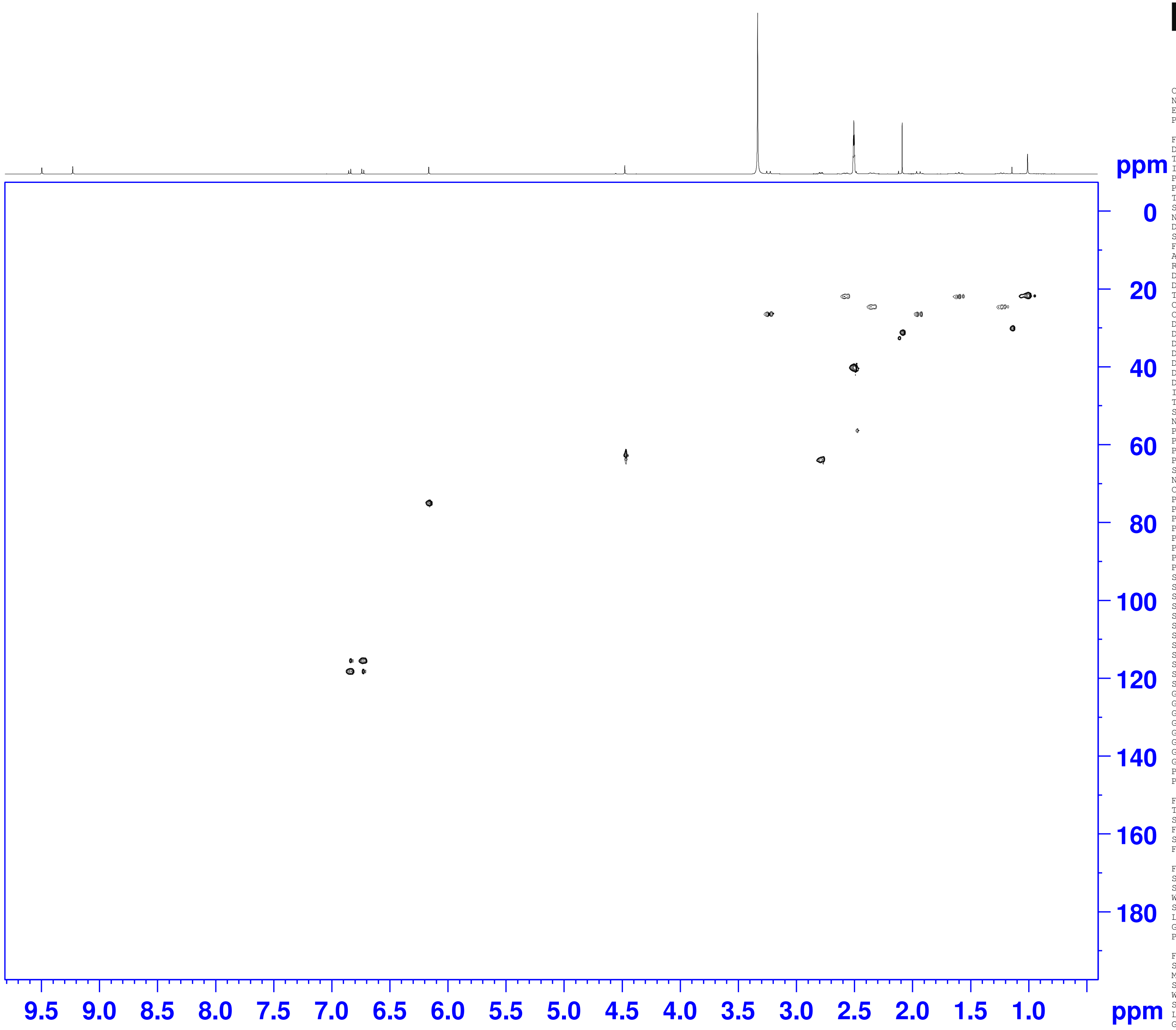


```
Current Data Parameters
NAME      ppa19_21978Fa
EXPNO                 6
PROCNO                1

F2 - Acquisition Parameters
Date_          20210718
Time              23.24 h
INSTRUM           spect
PROBHD   Z113652_0281 (
PULPROG  hsqcedetgpsisp2.3
TD                 2048
SOLVENT            DMSO
NS                   24
DS                   32
SWH            4708.098 Hz
FIDRES         4.597752 Hz
AQ            0.2174976 sec
RG                 1820
DW              106.200 usec
DE                13.32 usec
TE                298.0 K
CNST2       145.0000000
CNST17       -0.5000000
D0           0.00000300 sec
D1           1.50000000 sec
D4           0.00172414 sec
D11          0.03000000 sec
D16          0.00020000 sec
D21          0.00344800 sec
D24          0.00086200 sec
IN0          0.00001940 sec
TDav                  1
SFO1        500.2825557 MHz
NUC1                 1H
P1                 8.96 usec
P2                17.92 usec
P28             1000.00 usec
PLW1        25.00300026 W
SFO2        125.8074566 MHz
NUC2                13C
CPDPRG[2          garp4
P3                 9.00 usec
P14              500.00 usec
P24             2000.00 usec
P31             1895.00 usec
PCPD2             70.00 usec
PLW0                  0 W
PLW2        86.00000000 W
PLW12        1.42159998 W
SPNAM[3] Crp60,0.5,20.1
SPOAL3            0.500
SPOFFS3               0 Hz
SPW3        10.64299965 W
SPNAM[7]    Crp60comp.4
SPOAL7            0.500
SPOFFS7               0 Hz
SPW7        10.64299965 W
SPNAM[18  Crp60_xfilt.2
SPOAL18           0.500
SPOFFS18              0 Hz
SPW18        2.56369996 W
GPNAM[1]     SMSQ10.100
GPZ1              80.00 %
GPNAM[2]     SMSQ10.100
GPZ2              20.10 %
GPNAM[3]     SMSQ10.100
GPZ3              11.00 %
GPNAM[4]     SMSQ10.100
GPZ4              -5.00 %
P16             1000.00 usec
P19              600.00 usec

F1 - Acquisition parameters
TD                  256
SFO1           125.8075 MHz
FIDRES       201.353088 Hz
SW              204.862 ppm
FnMODE    Echo-Antiecho

F2 - Processing parameters
SI                 1024
SF          500.2800000 MHz
WDW               QSINE
SSB                   2
LB                    0 Hz
GB                    0
PC                 1.40

F1 - Processing parameters
SI                 1024
MC2       echo-antiecho
SF          125.7955061 MHz
WDW               QSINE
SSB                   2
LB                    0 Hz
GB                    0
```
