# Supplementary material for: ent-Clavilactone J and Its Quinone Derivative, Meroterpenoids from the Fungus Resupinatus sp
Source: J Nat Prod. 2023 Nov 6;86(11):2580–4. doi: 10.1021/acs.jnatprod.3c00174 (PMC10683060; doi:10.1021/acs.jnatprod.3c00174)
Supplement: Supplementary file 2 — np3c00174_si_002.zip [file np3c00174_si_002.zip › ent-clavilactone J in DMSO ppa19_21978Fa/7 13C/pdata/1/email_ppa19_21978Fa_7_1.pdf]

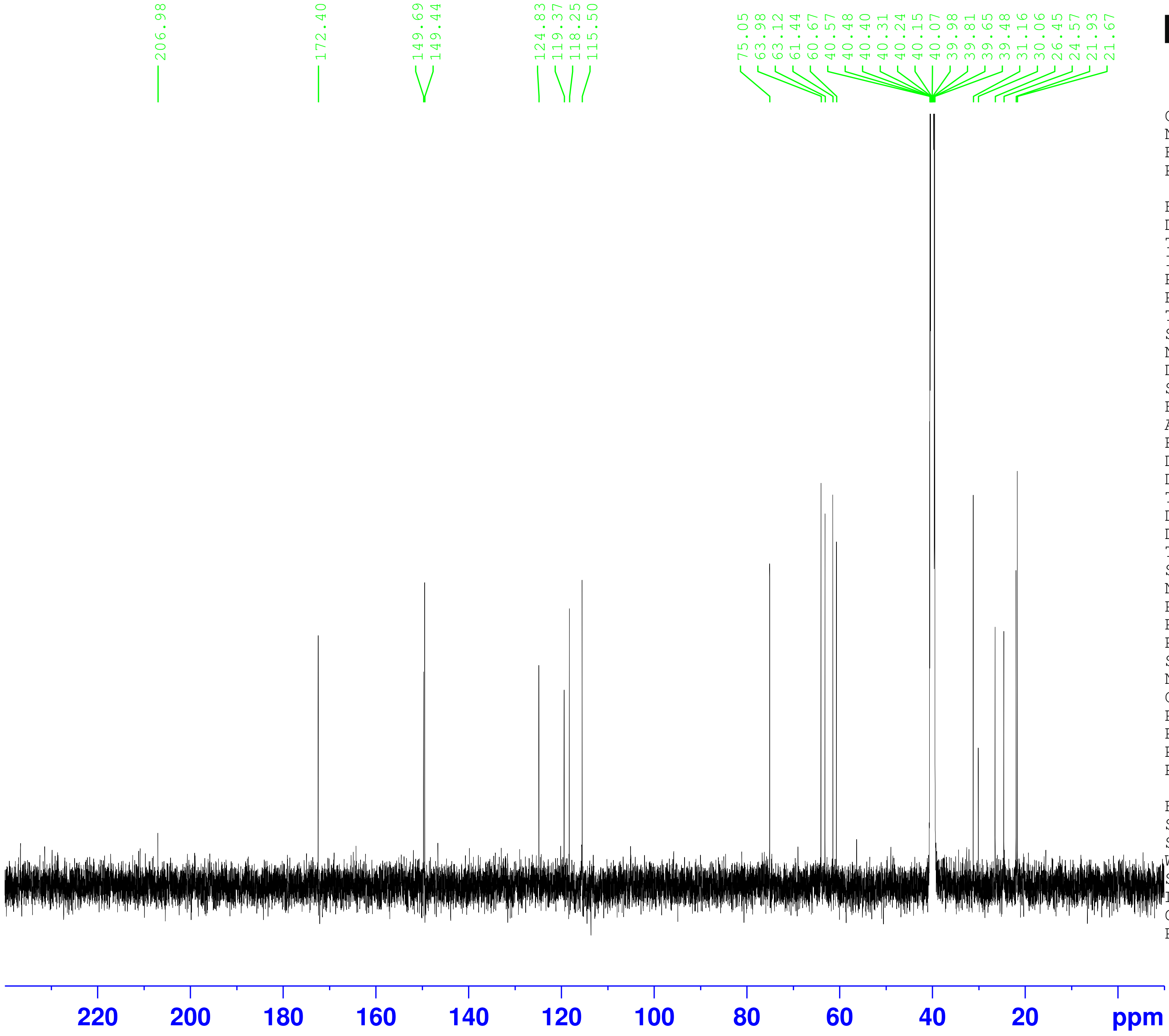

Current Data Parameters

NAME	ppa19_21978Fa	
EXPNO	7	
PROCNO	1	

F2 - Acquisition Parameters

Date_	20210719	
Time	7.09	h
INSTRUM	spect	
PROBHD	Z113652_0281 (	
PULPROG	zgpg30	
TD	65536	
SOLVENT	DMSO	
NS	8400	
DS	4	
SWH	33482.145	Hz
FIDRES	1.021794	Hz
AQ	0.9786710	sec
RG	310.97	
DW	14.933	usec
DE	6.50	usec
TE	298.0	K
D1	1.00000000	sec
D11	0.03000000	sec
TD0	1	
SFO1	125.8098467	MHz
NUC1	13C	
P0	3.00	usec
P1	9.00	usec
PLW1	86.00000000	W
SFO2	500.2820011	MHz
NUC2	1H	
CPDPRG[2	waltz65	
PCPD2	80.00	usec
PLW2	25.00000000	W
PLW12	0.35253999	W
PLW13	0.17732000	W

F2 - Processing parameters

SI	32768	
SF	125.7955061	MHz
WDW	EM	
SSB	0	
LB	1.00	Hz
GB	0	
PC	1.40	
